# The Biodiversity and Geochemistry of Cryoconite Holes in Queen Maud Land, East Antarctica

**DOI:** 10.3390/microorganisms7060160

**Published:** 2019-06-01

**Authors:** Stefanie Lutz, Lori A. Ziolkowski, Liane G. Benning

**Affiliations:** 1 GFZ German Research Centre for Geosciences, Telegrafenberg, 14473 Potsdam, Germany; 2 University of South Carolina, School of the Earth, Ocean and Environment, 701 Sumter St., EWS 617, Columbia, SC 29208, USA; lziolkowski@geol.sc.edu; 3 Department of Earth Sciences, Free University of Berlin, 12249 Berlin, Germany

**Keywords:** Cryoconite holes, Antarctica, high-throughput sequencing, bacteria, eukaryotes, carbon, ^13^C, ^14^C

## Abstract

Cryoconite holes are oases of microbial diversity on ice surfaces. In contrast to the Arctic, where during the summer most cryoconite holes are ‘open’, in Continental Antarctica they are most often ‘lidded’ or completely frozen year-round. Thus, they represent ideal systems for the study of microbial community assemblies as well as carbon accumulation, since individual cryoconite holes can be isolated from external inputs for years. Here, we use high-throughput sequencing of the 16S and 18S rRNA genes to describe the bacterial and eukaryotic community compositions in cryoconite holes and surrounding lake, snow, soil and rock samples in Queen Maud Land. We cross correlate our findings with a broad range of geochemical data including for the first time ^13^C and ^14^C analyses of Antarctic cryoconites. We show that the geographic location has a larger effect on the distribution of the bacterial community compared to the eukaryotic community. Cryoconite holes are distinct from the local soils in both ^13^C and ^14^C and their isotopic composition is different from similar samples from the Arctic. Carbon contents were generally low (≤0.2%) and older (6–10 ky) than the surrounding soils, suggesting that the cryoconite holes are much more isolated from the atmosphere than the soils.

## 1. Introduction

Cryoconite holes are water-filled depressions on ice surfaces caused by the preferential heating of accumulated low-albedo particles (e.g., cells, dust, and minerals) and subsequent localized melting into the surrounding ice [1]. They are oases of microbial diversity and host a consortium of microorganisms including photoautotrophic and heterotrophic prokaryotes, viruses, algae and other micro-eukaryotes [2,3,4,5]. Cryoconite holes are thought to be hotspots for biogeochemical cycling on glaciers and ice sheets [6,7,8].

In contrast to the Arctic, where cryoconite holes are ‘open’, in Antarctica they are often ‘lidded’ (frozen surface layer) or completely frozen [9]. Thus, they represent ideal systems for the study of microbial community assemblies since individual cryoconite holes can be isolated from the atmosphere for years. Nevertheless, melting can occur underneath the lid for up to twelve weeks in the austral summer, which enables microbial activity [10]. Compared to the Arctic, the Antarctic continent is characterized by lower temperatures and stronger katabatic winds. In these conditions, cryoconite holes represent ideal refuges for microbial life. In years of high melting, they can seed the surrounding environments.

Furthermore, cryoconite holes constitute an important carbon source for local glacial microbes as well as downstream communities [6]. The cryoconite material is thought to consist of a mixture of locally produced microbial carbon as well as allochthonous inputs [11]. However, the relative contributions of microbially derived carbon, inorganic carbon from mineral dusts or C from combustion products to Antarctica glaciers are currently unknown. Owing to its geographic isolation, Antarctica experiences considerably lower levels of anthropogenic C inputs and therefore represents an ideal system to explore carbon accumulation in a pristine environment afar from anthropogenic activities. This is particularly important since it has been shown that the accumulation of cryoconite material results in the darkening of glacial surfaces and hence leads to higher glacial melt rates [12,13]. Comparing carbon accumulations from the northern and southern hemisphere will enable us to determine the contribution of humans to the darkening of glaciers and ice sheets.

Whilst microbial communities have been extensively studied in the McMurdo Dry Valleys [9,14,15], the Queen Maud Land is more understudied in terms of its biodiversity. A few studies have been published on terrestrial and aqueous microbial communities [16,17,18], yet, those data on cryoconite holes are scarce. Moreover, to our knowledge, the application of radioactive labelling to study microbial activity has been absent so far in the Utsteinen, Dubois and Petrelnuten areas of Queen Maud Land, and thus, these locations can be regarded as pristine and suitable for using natural abundance radiocarbon measurements.

Here, we investigate the bacterial and eukaryotic community compositions using high-throughput sequencing of the 16S and 18S ribosomal small subunit genes, respectively. We document biogeographic patterns by comparing the microbial assemblages in cryoconite holes from four sampling locations in Queen Maud Land and from surrounding lake, snow, soil and rock sample in order to explore potential external inputs to the community compositions. Furthermore, we cross-correlate the molecular findings with a broad range of geochemical analyses of the cryoconite ice and sediments. Our aim is to assess (1) how the cryoconite hole communities are spatially structured; (2) which parameters shape their community structures; (3) whether similar patterns for bacteria and eukaryotes exist; as well as (4) the age and source(s) of the carbon and its bioavailability potential. 

We present data from the first combined molecular microbial, carbon isotopic and broader geochemical study of more pristine cryoconite holes in East Antarctica, which are generally less affected by anthropogenic activities compared to those in coastal Antarctic regions or in Arctic or Alpine settings. 

## 2. Materials and Methods

### 2.1. Field Sites and Sampling

**Figure 1:**
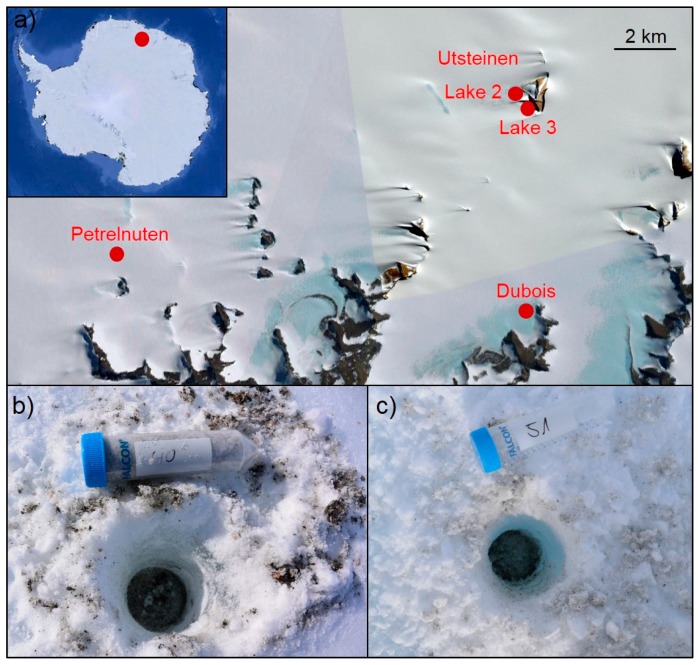
The four sampling locations in East Antarctica comprising 35 samples (**a**) and two representative images of the sampled cryoconite holes near Lake 3 (**b**) and Lake 2 (**c**) in Utsteinen.

A total of twenty-four cryoconite hole samples were collected in Queen Maud Land between the 31^st^ of January and 8^th^ of February in 2017 (Figure 1 and Table 1) near the Utsteinen Nunatak (“Lake 2” and “Lake 3”), in Dubois and in Petrelnuten (see Table 1 for GPS coordinates). Wind direction in the area is generally governed by the katabatic winds that blow from the plateau (south) towards the coast.

All cryoconite holes were ice-lidded, i.e., they were frozen and covered by an ice layer of approximately 10 cm. They were on average 5–10 cm in diameter. The sediment layer was at about 20 cm depth and of 3–5 cm thickness. There was an air pocket between the sediment and ice layer. A Kovacs ice drill was used to remove the ice layer and the frozen cryoconite hole sediment was excavated through the drilling and scooped from the surface debris into the tubes using a sterilized ice scoop. The Kovacs drill was cleaned off through drilling into clean ice between cryoconite holes.

To investigate potential sources of the microorganisms in the cryoconite holes we also collected a total of eleven samples from different surface habitats nearby for DNA sequencing. These comprised five soil, one snow, three endoliths and two lake samples. Soil and endolith samples were collected using a sterilized scoop or a hammer and chisel and filled into sterile 50 mL centrifuge tubes or sterile sampling bags. The lakes were completely frozen and an approximately 70–80 cm thick ice layer had to be penetrated using a Kovacs ice drill to reach liquid lake water. Lake water was welling up and was sampled into sterile 1 L plastic bottles using a sterilized turkey baster. For the snow sample, several sterile large sampling bags were filled using a sterilized scoop and slowly thawed on-site at ambient temperatures (~5–10 °C). Lake (1 L) and snow melt (20 L) water were filtered through sterile Nalgene single-use filtration units (pore size 0.22 μm) and filters were stored frozen. All samples were transported back to the home lab (GFZ Potsdam and University of South Carolina) frozen and stored at −80 °C (DNA samples) or −20 °C (all other samples) analyzed.

### 2.2. DNA Sequencing and Bioinformatics

DNA was extracted from the cryoconite hole sediments and soils using the PowerSoil^®^ DNA Isolation kit (MoBio Laboratories). DNA stored on filters (lakes, snow) was extracted using the PowerWater^®^ DNA Isolation kit (MoBio Laboratories). The 16S and 18S rRNA amplicons were prepared according to the Illumina “16S Metagenomic Sequencing Library Preparation” guide [19] and as described previously [20]. In brief, 16S rRNA genes were amplified using the bacterial primers 341F (5’-CCTACGGGNGGCWGCAG) and 785R (5’-GACTACHVGGGTATCTAATCC) spanning the V3–V4 hypervariable regions. The 18S genes were amplified using the primers 528F (5’ GCGGTAATTCCAGCTCCAA) and 706R (5’ AATCCRAGAATTTCACCTCT) [21] spanning the V4-V5 hypervariable regions. The pooled amplicon library was sequenced on the Illumina MiSeq using paired 300-bp reads at the University of Bristol Genomics Facility (Table S1). Quality filtering of the sequences was carried out as described in Lutz et al. [20] and included trimming of low quality 3’ ends, joining of paired sequences, removal of sequences below a minimum Phred quality score of Q20 and exclusion of chimeric sequences. Qiime [22] was used for the following processing steps. Operational taxonomic units (OTU) were picked de novo and clustered at 97% and 99% similarity for 16S and 18S, respectively. A stricter threshold is required for 18S sequences due to more closely related species in particular within the green algae. Taxonomic identities were assigned using the databases Greengenes [23] for 16S and Silva [24] for 18S. Singletons were removed from both and sequences matching chloroplast DNA from the 16S data set prior to further analyses. OTU tables were rarefied to 58,000 and 26,000 reads per sample for the 16S and 18S data sets, respectively. Core 16S and 18S OTUs across samples were computed using the available Qiime script. Representative sequences of the identified OTUs were submitted to NCBI to verify the closest hits using BLAST [25]. OTU frequencies were compared across sampling locations using ANOVA to identify statistically significant differences between the OTU abundances. OTU tables were imported into R (v.3.2.3) for the creation of community compositional bar charts. SourceTracker (v.0.9.5) [26] was used to predict the source of the bacterial and eukaryotic communities using the cryoconite hole samples as sinks and lake, soil, endolith and snow samples as potential sources of microbial inputs.

Raw sequences have been deposited in the SRA under accession number PRJNA529498.

### 2.3. Organic Geochemistry

Aliquots of the cryoconite hole solids were dried in an oven at 105 °C for one week and subsequently homogenized and milled to a fine powder using an agate mortar and pestle. Total carbon (TC), total organic carbon (TOC), total nitrogen (TN) and total organic nitrogen (TON) were determined using an EUROEA3000 elemental analyzer (EuroVector). For TC and TN approximately 6 mg of sample material were transferred into Sn-capsules and acetanilide was used for calibration. For TOC and TON approximately 15 mg of each sample were loaded into in Ag capsules and urea was used as a calibration standard.

Thermovaporization (at 300 °C) and open system pyrolysis (between 300 and 600 °C) were carried out on a Quantum MSS-2 ThermalAnalyser© interfaced with an Agilent GC 6890A gas chromatograph as described in [27], in order to gain some additional insights into the nature of any present organic compounds. 

Furthermore, we used thermogravimetric analyses (TGA, Mettler Toledo TGA/DSC1 Thermal Analyzer, 10°/min ramp between 30 and 1000 °C) to assess the mass loss, which is a measure of the contents of water and other volatile (carbon, sulfur, etc.) species in our dried cryoconite solids.

Other aliquots of the solids from cryoconite holes and soils were prepared for stable carbon isotope (^13^C) and radiocarbon (^14^C) analysis. Freeze-dried and mortar and pestle homogenized material was transferred into pre-baked quartz tubes and combustion chemicals were added (cupric oxide and silver wire). The quartz tubes were evacuated on a vacuum line, before being flame sealed and combusted at 900 °C for 2 hours. The generated CO_2_ was then purified and quantified before being sealed into Pyrex tubes and shipped to the University of Georgia Center for Isotope Analysis, where samples were graphitized and the ^14^C was measured via accelerator mass spectrometry (AMS). Before graphitization, a subsample of the CO_2_ was taken for ^13^C analysis using a stable isotope ratio mass spectrometer. ∆^14^C values for all samples were normalized to δ^13^C values of −25 ‰ to correct for isotope fractionation and presented as ∆^14^C (‰) relative to the standard NSB Oxalic Acid (NIST-SRM-4990C) [28]. Uncertainties of the isotope measurements are ±0.1 ‰ for δ^13^C and ±5 ‰ for ∆^14^C.

### 2.4. Inorganic Geochemistry

X-ray fluorescence (XRF) analyses were used to determine the elemental composition of the solid samples using a PANalytical AXIOS Advanced XRF machine equipped with a rhodium tube. The measurements were calibrated using 130 standards made of different materials including basalts, granites and soil sediments (e.g., JSO-1, JSO-2, GXR-2, GXR-3, GXR-4, GXR-5, GXR-6). Approximately 1 g of each sample, 6 g of di-Lithiumtetraborate (FX-X65-2) and 0.5 g of ammonium nitrate were melted into glass disks. 

Aliquots of the homogenized solid powders from each sample were also analyzed by powder X-ray diffraction (XRD; Bruker D8diffractometer; CuKa1; 530º2y; 0.105º2y/step) to determine their mineralogical composition.

### 2.5. Aqueous Geochemistry

After thawing of the frozen cryoconite hole material, the meltwater derived from the ice fractions within the samples were filtered through 0.22 μm single-use syringe filters (Millipore). Samples were analyzed in duplicates by ion chromatography (IC) with conductivity detection (ICS 3000, Dionex Corp.). The analytical column AS 11 HC (Dionex Corp.) was used at a temperature of 35 °C for chromatographic separation of the anions. A KOH solution at a flow of 0.38 mL/min and varying concentration over time was used to elute the compounds. Standards comprising all investigated compounds were used for quantification of the inorganic anions (F^−^, Cl^−^, SO_4_^2−^, NO_3_, PO_4_^3−^) and organic acids (formic acid, acetate, propionate, butyrate, valerate, oxalate).

### 2.6. Statistical Analyses

Principal component analysis (PCA) and (partial) canonical correspondence (CCA) analyses were performed using the rda and cca functions of the R (v.3.3.2) ‘vegan’ package, respectively. The input consisted of the rarefied and square-root transformed species abundance table containing OTUs with a minimum relative abundance of 1%. For CCA, all data from the XRF (elemental composition), IC (aqueous chemistry) and TN/TC analyses were used as predictor variables, which were normalized by square root transformation. Variance inflation factors (VIF) were computed to check for redundancy in the predictor variables, which were then subsequently excluded from the analysis until all VIFs were below 10. A conditioning matrix comprising sample coordinates and elevation was incorporated for partial CCA and to test for the significance of spatial effects using 999 permutations. In addition, PERMANOVA was carried out in R using the vegdist function (method = ”bray”) and the adonis2 function (permutations = 999) to test for the significance and contribution of the geographic locations on the distribution of community compositions.

## 3. Results

### 3.1. Bacterial Community Composition

**Figure 2:**
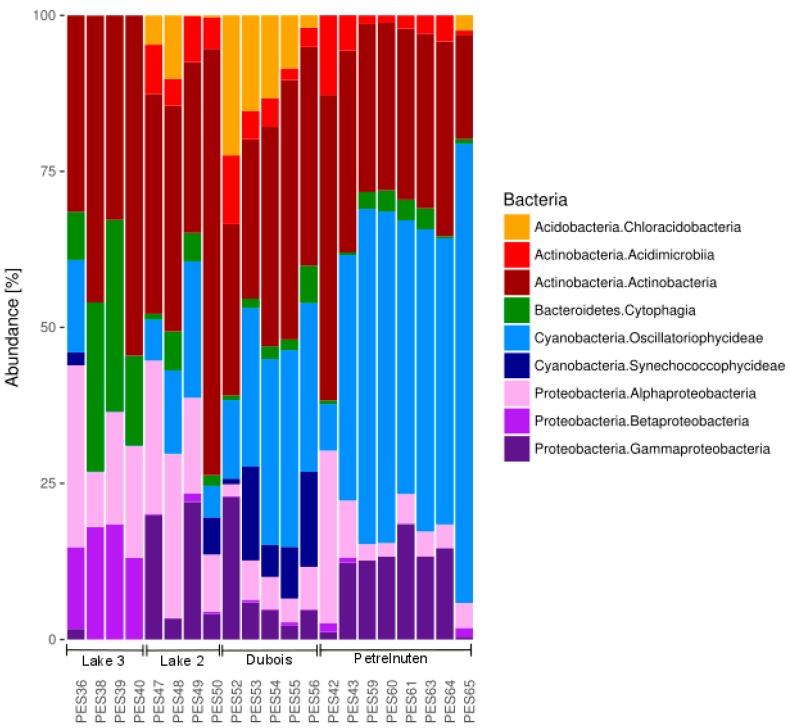
The bacterial community composition of all cryoconite hole samples with the nine most abundant bacterial classes comprising >99% of the total community composition. Full details can be found in Table S2.

**Figure 3:**
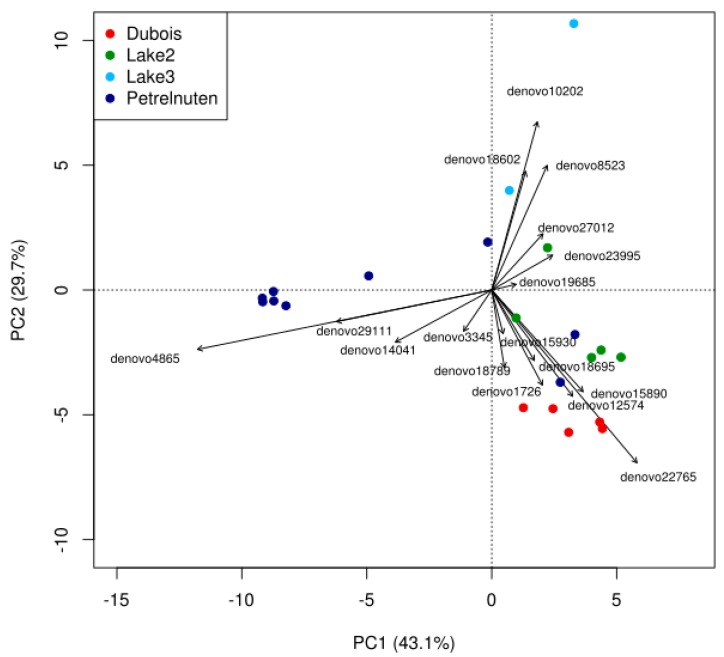
Principal component analysis of bacterial OTUs revealing compositional differences between sampling locations. The lowest possible level of taxonomic identification varied strongly between OTUs and in the majority of cases could only be resolved down to the family level. Full details of the OTU identities can be found in Table S4.

A total of 3,015,821 sequences passed the quality control and were assigned to bacterial taxa (Table S1). Proteobacteria, Cyanobacteria and Actinobacteria made up the largest proportion (Figure 2, Table S2). Samples clustered according to the four different sampling sites Lake 2, Lake3, Dubois and Petrelnuten (Figure 3). Photosynthetic cyanobacteria were by far more abundant in Dubois and Petrelnuten compared to Lake 2 and Lake 3. Cyanobacteria were mostly represented by Oscillatoriophycideae (i.e., Xenococcaceae, Phormidiaceae) with minor contributions of Synechococcophycideae (i.e., Pseudanabaenaceae), mostly in Dubois. Alphaprotebacteria (i.e., Sphingomonadaceae) and Gammaproteobacteria (i.e., Xanthomonadaceae) were more abundant near Lake 3, whereas Betaproteobacteria (i.e., Comamonadaceae) were more abundant in Lake 2 and Petrelnuten. In general, the majority of the OTUs could not be assigned down to the species level and often the genus (~28%) or even the family (~50%) level were the highest taxonomy levels the OTUs could be attributed to. Archaea could only be detected in one sample (PES48) in very low abundance (0.002%) and were identified as *Nitrososphaera* (data not shown).

The principal component analysis (PCA, Figure 3, Data S1) revealed that the taxa causing the separation by location are the cyanobacterial family of the Xenococcaceae (OTU denovo4865), which showed a higher abundance in Petrelnuten, and the cyanobacterial genus *Phormidium* (OTU denovo22765), which showed a higher abundance in Dubois. In addition, cryoconite samples near Lake 3 were characterized by higher abundance of the genus *Hymenobacter* within the Bacteroidetes (OTU denovo3345), the family Nocardioidaceae within the Actinobacteria (OTU denovo18602) and the family Comamonadaceae within the Betaproteobacteria (OTU denovo8523). A PERMANOVA analysis revealed that geographic location is significantly (*p* < 0.001) affecting the distribution of the bacterial community composition and that it explains ~59% of the variation (R^2^ = 0.58616).

Furthermore, when comparing samples across habitats, PCA revealed that the community composition within the cryoconite habitats is more similar compared to the other habitats (i.e., soils, lakes, endoliths, and snow, Figure S1). The separation is characterized by a higher abundance of OTU denovo19799 in non-cryoconite samples, which corresponds to the unclassified Acidobacteriaceae Ellin6075 (Acidobacteria).

The core microbiome revealed that across samples shared OTUs mostly correspond to Actinobacteria families, the *Sphingomonas* genus within the Alphaproteobacteria and the cyanobacterial family of Xenococacceae (Table S3).

**Figure 4:**
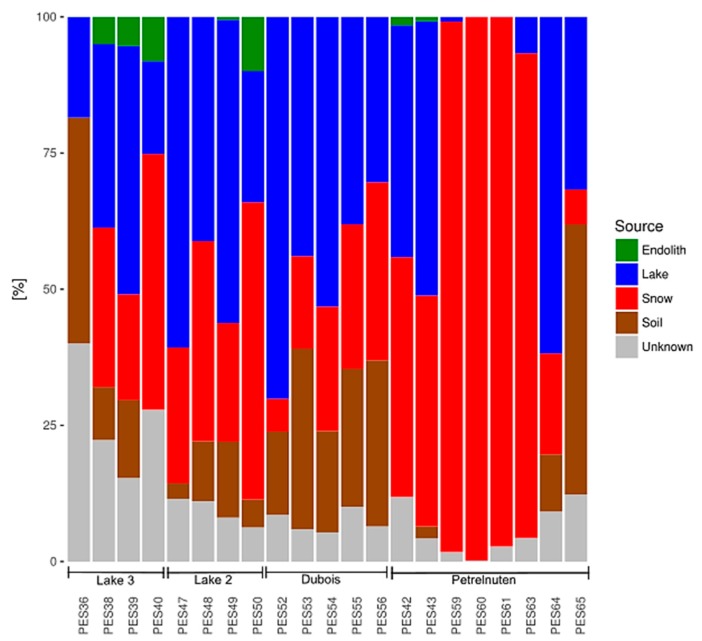
The relative contributions of the habitats endolith/rock, lake, snow and soil as potential sources for the composition of the bacterial community in the cryoconite hole samples, as well as the proportion from other (unknown) sources. Potential sources are more similar within the same sampling locations.

The results of the SourceTracker analysis suggest that the potential sources for the microbial communities in the cryoconite holes differ between the four locations. In the Utsteinen (Lake 2 and 3) and Dubois samples there are approximately equal contributions from the snow and lake samples (Figure 4). Soil as source is more pronounced in the Dubois area, whereas there are more unknown sources in the cryoconite samples near Lake 2 and 3. The Petrelnuten samples suggest snow as the predominant source. Across all samples endoliths/rocks play a minor role as potential sources.

### 3.2. Eukaryotic Community Composition

**Figure 5:**
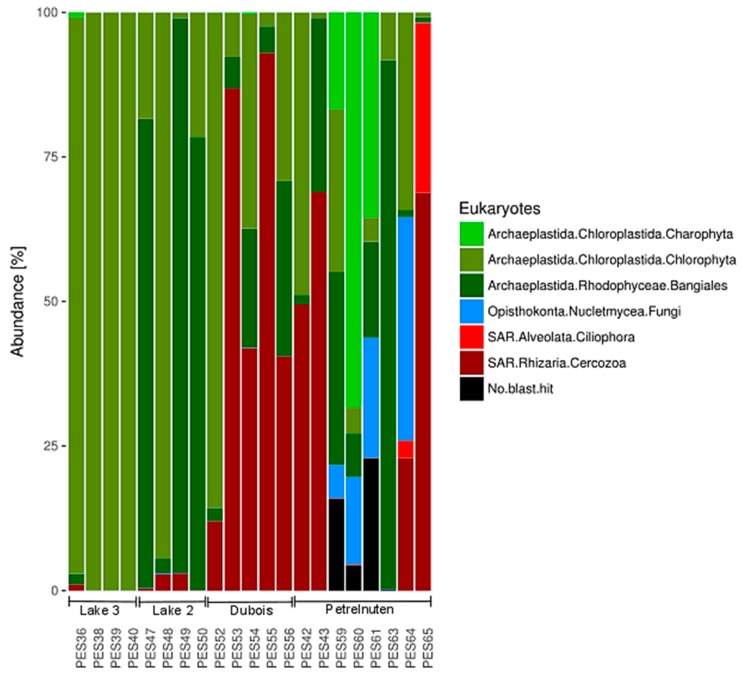
The eukaryotic community composition of all cryoconite hole samples. Full details can be found in Table S5.

**Figure 6:**
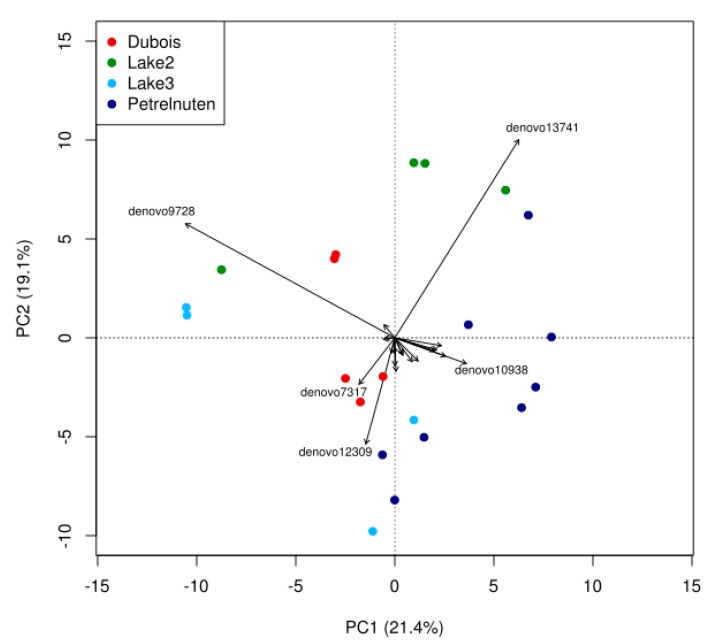
Principal component analysis of eukaryotic OTUs revealing compositional differences between sampling locations. The lowest possible level of taxonomic identification varied strongly between OTUs. Full details of the OTU identities can be found in Table S7.

A total of 2,231,258 sequences passed the quality control and were assigned to eukaryotic taxa (Table S1). The Utsteinen samples (Lake 2 and 3) mostly consisted of green algae belonging to Chlorophyta and red algae belonging to Bangiales (Figure 5, Table S5). A higher proportion of the Cercozoa (Rhizaria) could be found in Dubois, whereas fungi could almost only be detected in samples from Petrelnuten. A PERMANOVA analysis revealed that geographic location is significantly (*p* < 0.001) affecting the distribution of the eukaryotic community composition, yet it only explains ~36% (R^2^ = 0.35969) of the variation. In addition, the PCA analysis revealed no clear pattern for the distribution of the eukaryotic communities (Figure 6, Data S2). 

The core microbiome consists of solely two OTUs corresponding to *Chlorella* sp. (Chlorophyta) and *Pyropia fallax* (Bangiales) (Table S6).

Furthermore, when comparing samples across habitats, PCA revealed that the community composition within the cryoconite habitats is more similar compared to the other habitats (i.e., soils, lakes, endoliths, and snow, Figure S2).

**Figure 7:**
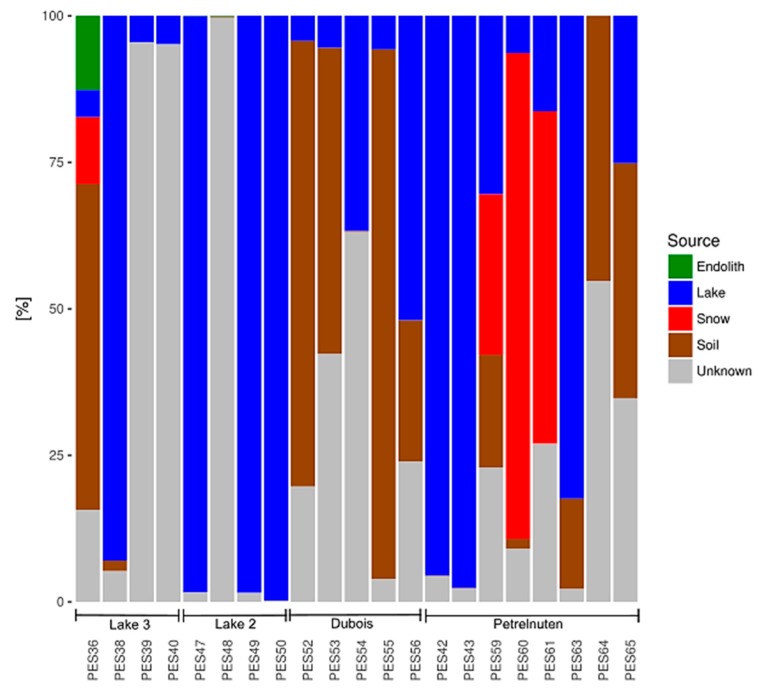
The relative contributions of the habitats endolith/rock, lake, snow and soil as potential sources for the composition of the eukaryotic community in the cryoconite hole samples, as well as the proportion from other (unknown) sources. The distribution of the potential sources does not follow any trends within the four sampling locations and seems to be randomly distributed.

The predicted sources varied immensely for the samples within one location and were mostly attributed to either the lakes or to unknown sources (Figure 7). Snow as a potential source was more abundant in the Petrelnuten samples, whereas soils played a more important role in all samples from Dubois. Samples containing a high abundance of Bangiales were predicted to predominantly derive from lake water samples. In contrast, samples with a high abundance of Chlorophyta (e.g., PES38, PES39, PES40) seemed to be mostly influenced by unknown sources. 

### 3.3. Organic Geochemistry

All samples were characterized by very low carbon and nitrogen contents (Table S8). Total carbon and total organic carbon were below 0.2% and total nitrogen and total organic nitrogen either below or just at the detection limit of our analyses (0.01%). These patterns were corroborated by the TGA analysis, which revealed a maximum mass loss below 1% across all samples (Table S8). Similarly, thermovaporization did not result in a significant signal, suggesting that the presence of live organic matter was very low, while the higher temperature open pyrolysis only revealed very low signals for mainly aromatic compounds and short or cyclic alkanes (Table S8).

Cryoconite sediments were more enriched in ^13^C (δ^13^C = −16.7 ± 3.1‰, n = 5) than soil samples (δ^13^C = −31.4 ± 5.4 ‰, n = 5). On the other hand, cryoconite hole samples were also far more depleted in radiocarbon (∆^14^C = −548 ± 131 ‰) than the soil samples (∆^14^C = −116 ± 65 ‰). These average radiocarbon values correspond to 6700 ^14^C years for cryoconite and ~1000 ^14^C years for soils. Therefore, these results indicate that the carbon in cryoconite hole sediments were isotopically distinct from the soils with respect to both the stable carbon and radiocarbon isotopic compositions.

### 3.4. Inorganic Geochemistry

The major elemental compositions of the solids as analyzed by X-Ray fluorescence showed no distinguishable differences, while the trace elemental compositions showed only minor variations, with slightly higher amounts of Ba in Petrelnuten, Sr and V in Dubois and Zr at Lake 2 (Figure S3, Table S9). 

Similarly, the mineralogy across all samples revealed typical minerals found in granites and gneisses including quartz and plagioclase with minor contribution of biotite, pyroxene, and amphibole. 

Most aqueous chemical concentrations were low and revealed no trends among the four sampling locations. NO_3_^-^ ranged between only 0 and 2.25 µgL^−1^, whereas PO_4_^3−^ was mostly below the detection limit, except in the case of three samples from Petrelnuten, which ranged between 0.25 and 0.85 µgL^−1^ (Table S10).

### 3.5. Relationship Between Microbial Community Compositions and Geochemistry

In order to identify parameters shaping the microbial community compositions, all geochemical components were included in the canonical correspondence analysis (CCA). Most predictor variables showed high co-linearity and were subsequently excluded in the CCA in a stepwise process until all VIFs were below 10. 

For the bacterial community composition, only the aqueous components NO_3_^−^, SO_4_^2−^, Cl and solid TC remained and could explain 49% of the total variance (Figure S4). The model of the partial CCA with GPS coordinates and elevation included as a conditioning matrix, could explain 38%, thus suggesting that 11% was derived from spatial effects. The samples from Lake 3 clustered with higher nitrate concentrations and from Dubois with a higher TC abundance. Permutational ANOVA revealed that NO_3_^−^ (*p* = 0.005), SO_4_^2−^ (*p* = 0.03), and Cl^−^ (*p* = 0.018) were significantly related to the bacterial community composition. 

For the eukaryotic community composition, again the aqueous components NO_3_^−^ and Cl^−^, and solid TC, but also Fe_2_O_3_, Na_2_O, Cr and Ni remained and could explain 70% of the total variance. The model of the partial CCA with GPS coordinates and elevation included as a conditioning matrix, could explain 51%, thus suggesting that 19% was derived from spatial effects. However, permutational ANOVA revealed that none of the variables was significantly related to the eukaryotic community composition. 

## 4. Discussion

We show that the geographic location has a higher impact on the distribution of the bacterial compared to the eukaryotic communities. This is in contrast to the findings of Sommers et al. [14] in ice-lidded cryoconite holes in the McMurdo Dry Valleys of Antarctica where the eukaryotic distribution mirrored that of bacteria. 

The relative abundance of bacterial taxa in our samples clustered according to locations. This is on par with previous studies, which found that the community compositions were similar between cryoconite holes sampled in close proximity to each other [29,30,31]. Such a pattern suggests local sources for the bacteria and/or similar environmental selection pressures within one location. 

At the class level, Cyanobacteria, Proteobacteria and Actinobacteria were overall most abundant in our samples, which matches previous findings from the McMurdo Dry Valleys [14,15]. One major difference is the lower abundance of Bacteroidetes in our samples. Although, Bacteroidetes (Cytophagia) were not abundant in all our samples, they were surprisingly the most abundant in the samples near Lake 3. Foreman et al. [9] found a high abundance of Bacteroidetes and more specifically Cytophagia and Flavobacteria in the sediment of their cryoconite holes from the McMurdo Dry Valleys, while in contrast to our findings, Cameron et al. [29] detected a higher abundance of Firmicutes, Deltaproteobacteria and Epsilonproteobacteria in their cryoconite hole samples from Signy Island and Princess Elisabeth Land. Overall a large number of OTUs could not be identified on the genus or species level, and thus, represents so far undiscovered diversity.

Webster-Brown et al. [15] found that the Antarctic cryoconite hole communities on glaciers generally reflect the surrounding source material of cryoconite. We did not find such dispersed cryoconite material outside the cryoconite holes since our sampling locations were not in the close vicinity of glaciers, but surrounded by large snow masses, frozen lakes or soils. The samples from Petrelnuten showed the highest similarity with the collected snow samples, which was freshly collected after a snowstorm. The similarity between the snow and the cryoconite material suggests that these samples are more affected by aeolian material from afar, in particular, since the cryoconite holes in Petrelnuten were exclusively located on the lee site of mountains. Aeolian transport has also previously been proposed to account for similarities over short distances in Antarctica [30]. 

In contrast, and not surprisingly, the samples from Lake 3 and Lake 2 seemed to receive bacterial contributions from the lake water, surrounding soil, as well as the snow. Soil played a more important role in Dubois, where there is no lake nearby. However, in particular in case of the samples collected on the shore of the frozen lakes, it is impossible to determine whether the lakes function as a source for the cryoconite holes, or whether the cryoconite microbiome feeds back into the lakes during high melt years [32].

The community compositions were not closely related to the geochemical parameters, apart from the dissolved nutrients in the cryoconite ice melt water (i.e., NO_3_^−^, SO_4_^2−^ and Cl^−^). None of the solid characteristics (i.e., elemental composition, type of bedrock) of the cryoconite sediment could explain the taxa distribution. An equivalent lack of relationships was described for Arctic cryoconites (e.g., Edwards et al. [33]) in which subtle abiotic variability resulted in only minor local variations of the bacterial community composition. 

In contrast to the bacteria, the geographic location could only explain ~36% of the variation in the eukaryotic communities and no potential sources of the eukaryotic taxa were evident despite the multitude of relationships tested. It is well known that wind is likely the dominant means in redistributing organic matter between the various local habitats including snow, ice, soils, lakes and cryoconite holes. The average larger cell sizes of eukaryotes may lead to shorter travel ranges and therefore lower dispersal rates than for bacteria [34].

The overall most abundant eukaryotic groups in all samples were phototrophic algae (Chlorophyta, Bangiales) and heterotrophic Rhizaria (Cercozoa). Fungi were present in low abundance with the exception of the samples from Petrelnuten. In addition to those groups other studies [4,14,30] have also found Rotifera, Tardigrada, Ciliophora and Ochrophyta in the McMurdo Dry Valleys, which were only present in our samples in low abundances. The higher abundance of phototrophic algae in both Utsteinen samples (Lake 3, Lake 2) could be due to the fact that they are in close vicinity to lakes. 

Several independent methods (i.e., elemental analysis, thermogravimetry, thermovaporization, pyrolysis) confirmed the very low abundance of organic carbon across all cryoconite hole samples (≤0.2%). Such low carbon and nitrogen contents are not surprising and comparable to values that Cameron et al. [29] found in samples from Signy Island (0.46%), whereas Arctic cryoconite holes are more abundant in carbon and at some locations contain over 10% organic carbon [2,11].

The ^13^C and ^14^C isotopic composition in the Antarctic cryoconite hole samples analyzed here were unique in comparison to Arctic cryoconites [11,35,36] or the surrounding soils (Table 2). Their stable isotopic composition (−12.9 to −20.9‰) was more enriched in ^13^C than the local soils (−25.2 to −30.6‰) with the soils being more akin to Arctic cryoconite hole materials (−23 to −30‰, [11,35,36]). Such a carbon isotopic composition is usually interpreted to be derived either from C4 plants or from inorganic carbonates. Due to the location of the sampling site, 200 km from coast in the inland of Antarctica, it is less likely that C4 plant material is an important contributor to the cryoconite material carbon. Based on our TC and TOC analyses (Table 2, Table S8) inorganic carbonate sources can also be excluded. The most probable explanation for these values is the fact that, in contrary to Arctic cryoconite holes, which are open during the summer melt season, the cryoconite holes samples from Queen Maud Land were sealed off by freezing. In addition, throughout the austral summer season the temperature at the sampling sites rarely if ever surpasses the melting point, and thus the cryoconites remained “lidded” or sealed off throughout the year and likely also over many years. Thus, it is possible that the initial heavier carbon (^13^C) was consumed leading ultimately to ^13^C values that appear to be enriched. 

This also matches the values for the ages that we determined. The carbon in our cryoconite samples was relatively old (6 to 10 ky) in comparison to the carbon in the nearby soils (0.5 to 2 ky). This difference in age of the carbon suggests that the systems are unique from each other, but also that the cryoconites have accumulated old carbon over time. While thermogravimetric analysis did not find any “living” cells in these samples, the carbon in these samples is likely of microbial origin since there are few other organic carbon sources in inland Antarctica. This is generally the case for most cryoconite holes in Antarctica as well as the Arctic [37,38].

If organic material was photosynthesized using the present-day atmosphere, the carbon in the cryoconite will be modern or young. Therefore, the only way these samples could contain such old carbon is by isolation from modern inputs for thousands of years (e.g., aged in place in a lidded environment) or the input of ancient carbon that is radiocarbon-free (e.g., fossil fuels). To explore the premise that these samples were influenced by anthropogenic inputs, we used an isotopic mass balance approach. If we assumed that the carbon in our samples were from one of two sources: modern biological inputs (recent atmosphere) or ancient inputs (>60 ky, the limit of radiocarbon dating), we find that that 55 + 13% of the organic carbon must be ancient carbon inputs. If we assumed that black carbon, or combusted carbon, from an equal mix of fossil (or ancient) and biological sources, such as the composition of the Asian brown cloud material [39], contributed in a large part to the TOC in our samples, than 37 ± 9% of the organic carbon must be from a 50/50 mix of old and young carbon. To our knowledge the only study to-date that has measured the ^14^C of black carbon on glaciers in the polar regions [40] found black carbon to be a small fraction of the total organic carbon pool (~5% of the organic carbon was black carbon) and was composed of young carbon (less than a thousand years old). Thus, based on our mass balance calculations, the fact that these cryoconites were ice-covered at the height of the austral summer, and the remote location where these samples were collected, it is unlikely that significant amounts of fossil fuel carbon (radiocarbon-free) material was accumulated in these cryoconites. Therefore, the most likely explanation is that the carbon has been isolated from the atmosphere for thousands of years and the carbon is being recycled within the closed cryoconite system. Unfortunately, ^13^C and ^14^C data for snow and lake samples are not available as not enough solid material was available for TC or isotopic analyses. 

The unique ^13^C and ^14^C composition of these Antarctic cryoconites may be due to their lidded nature. While Bagshaw et al. [41] suggested that the cryoconites in the Dry Valleys were only isolated from the atmosphere for only a few years, our data suggest that the cryoconites in the Sor Rondane Mountains in Queen Maude Land, may have become isolated from the atmosphere much longer ago. The combination of the stable isotopic values and ages both support the suggestion that the cryoconite holes in Queen Maude Land remained isolated from the atmosphere for a long time.

## Figures and Tables

**Table 1:** Overview of all samples collected in Usteinen (Lake 3, Lake 2), Dubois and Petrelnuten.

Sample ID	Location	Habitat	Collection Date	GPS Coordinates	Elevation
**PES36**	Lake 3	Cryoconite hole	31.01.2017	S 71.96345, E 23.31964	1342 m
**PES38**	Lake 3	Cryoconite hole	31.01.2017	S 71.96345, E 23.31964	1342 m
**PES39**	Lake 3	Cryoconite hole	31.01.2017	S 71.96345, E 23.31964	1342 m
**PES40**	Lake 3	Cryoconite hole	31.01.2017	S 71.96345, E 23.31964	1342 m
**PES42**	Petrelnuten	Cryoconite hole	31.01.2017	S 72.01292, E 22.82887	1492 m
**PES43**	Petrelnuten	Cryoconite hole	31.01.2017	S 72.01292, E 22.82887	1492 m
**PES47**	Lake 2	Cryoconite hole	01.02.2017	S 71.95867, E 23.31546	1316 m
**PES48**	Lake 2	Cryoconite hole	01.02.2017	S 71.95867, E 23.31546	1316 m
**PES49**	Lake 2	Cryoconite hole	01.02.2017	S 71.95867, E 23.31546	1316 m
**PES50**	Lake 2	Cryoconite hole	01.02.2017	S 71.95867, E 23.31546	1316 m
**PES51**	Lake 2	Cryoconite hole	01.02.2017	S 71.95867, E 23.31546	1316 m
**PES52**	Dubois	Cryoconite hole	01.02.2017	S 72.03793, E 23.29693	1361 m
**PES53**	Dubois	Cryoconite hole	01.02.2017	S 72.03793, E 23.29693	1361 m
**PES54**	Dubois	Cryoconite hole	01.02.2017	S 72.03793, E 23.29693	1361 m
**PES55**	Dubois	Cryoconite hole	01.02.2017	S 72.03793, E 23.29693	1361 m
**PES56**	Dubois	Cryoconite hole	01.02.2017	S 72.03793, E 23.29693	1361 m
**PES59**	Petrelnuten	Cryoconite hole	08.02.2017	S 72.01292, E 22.82887	1492 m
**PES60**	Petrelnuten	Cryoconite hole	08.02.2017	S 72.01292, E 22.82887	1492 m
**PES61**	Petrelnuten	Cryoconite hole	08.02.2017	S 72.01292, E 22.82887	1492 m
**PES62**	Petrelnuten	Cryoconite hole	08.02.2017	S 72.01292, E 22.82887	1492 m
**PES63**	Petrelnuten	Cryoconite hole	08.02.2017	S 72.01292, E 22.82887	1492 m
**PES64**	Petrelnuten	Cryoconite hole	08.02.2017	S 72.01292, E 22.82887	1492 m
**PES65**	Petrelnuten	Cryoconite hole	08.02.2017	S 72.01292, E 22.82887	1492 m
**PES4**	Utsteinen	Soil	19.01.2017	S 71.94535, E 23.34500	1359 m
**PES6**	Utsteinen	Soil	19.01.2017	S 71.94575, E 23.34525	1367 m
**PES33**	Dubois	Soil	30.01.2017	S 72.05169, E 23.25497	1352 m
**PES35**	Dubois	Soil	30.01.2017	S 72.04891, E 23.28334	1341 m
**PES44**	Petrelnuten	Soil	31.01.2017	S 72.01266, E 22.82781	1511 m
**PES57**	Utsteinen	Snow	02.02.2017	S 71.95177, E 23.34854	1362 m
**PES2**	Utsteinen	Endolith	18.01.2017	S 71.94535, E 23.34500	1359 m
**PES32**	Dubois	Endolith	30.01.2017	S 72.04891, E 23.28334	1341 m
**PES34**	Dubois	Endolith	30.01.2017	S 72.05169, E 23.25497	1352 m
**PES41**	Lake 3	Lake	31.01.2017	S 71.96589, E 23.33311	1315 m
**PES46**	Lake 2	Lake	31.01.2017	S 71.95818, E 23.31509	1317 m

**Table 2:** Overview of all ^13^C and ^14^C data of the cryoconite hole and soil samples and the corresponding ages of the carbon.

Sample ID	Location	Habitat	TC [%]	^13^C ‰	^14^C ‰	^14^C age, years BP
PES38	Lake 3	Cryoconite hole	0.02	−20.9	−638.4 ± 2.6	8170 ± 60
PES42	Petrelnuten	Cryoconite hole	0.10	−16.3	−537.0 ± 2.0	6190 ± 40
PES51	Lake 2	Cryoconite hole	0.03	−18.3	−408.9 ± 2.8	4220 ± 40
PES55	Dubois	Cryoconite hole	0.04	−14.9	−717.9 ± 1.6	10,160 ± 50
PES63	Petrelnuten	Cryoconite hole	0.12	−12.9	−435.8 ±1.9	4600 ± 30
PES4	Utsteinen	Soil	0.37	−28.2	−74.7 ±2.3	620 ± 20
PES6	Utsteinen	Soil	0.67	−25.2	−91.1 ±2.5	770 ± 20
PES33	Dubois	Soil	0.04	−30.6	−227.4 ± 3.5	2070 ± 40

## Supplementary Materials

The following are available online at https://www.mdpi.com/2076-2607/7/6/160/s1, Figure S1: Principal component analysis of the bacterial OTUs. Figure S2: Principal component analysis of the eukaryotic OTUs. Figure S3: Principal component analysis of selected trace elements. Figure S4: Partial canonical correspondence analysis of the bacterial abundances and all non-collinear environmental variables. Table S1: Number of sequences before and after quality control for bacterial and eukaryotic sequences. Table S2: Distribution of 97% clustered bacterial OTUs. Table S3: The core bacterial taxa present in all cryoconite hole samples. Table S4: Taxonomic identification of the discriminating OTUs in the principal component analysis. Table S5: Distribution of 99% clustered eukaryotic OTUs. Table S6: The core eukaryotic taxa present in all cryoconite hole samples. Table S7: Taxonomic identification of the discriminating OTUs in the principal component analysis. Table S8: Overview of solid total carbon, total organic carbon, total nitrogen, total organic nitrogen, thermogravimetry, thermovaporization and pyrolysis analyses. Table S9: Chemical composition of cryoconite sediments from X-ray fluorescence analysis. Table S10: Aqueous concentrations of nutrients and simple organics. Data S1: Output of the PCA of bacterial OTUs. Data S2: Output of the PCA of the eukaryotic OTUs.
