# Supplementary material for: The Biodiversity and Geochemistry of Cryoconite Holes in Queen Maud Land, East Antarctica
Source: Microorganisms. 2019 Jun 1;7(6):160. doi: 10.3390/microorganisms7060160 (PMC6616603; doi:10.3390/microorganisms7060160)
Supplement: Supplementary file 1 [file microorganisms-07-00160-s001.pdf]

Supplementary Information

The Biodiversity and Geochemistry of Cryoconite Holes in Queen Maud Land, East Antarctica

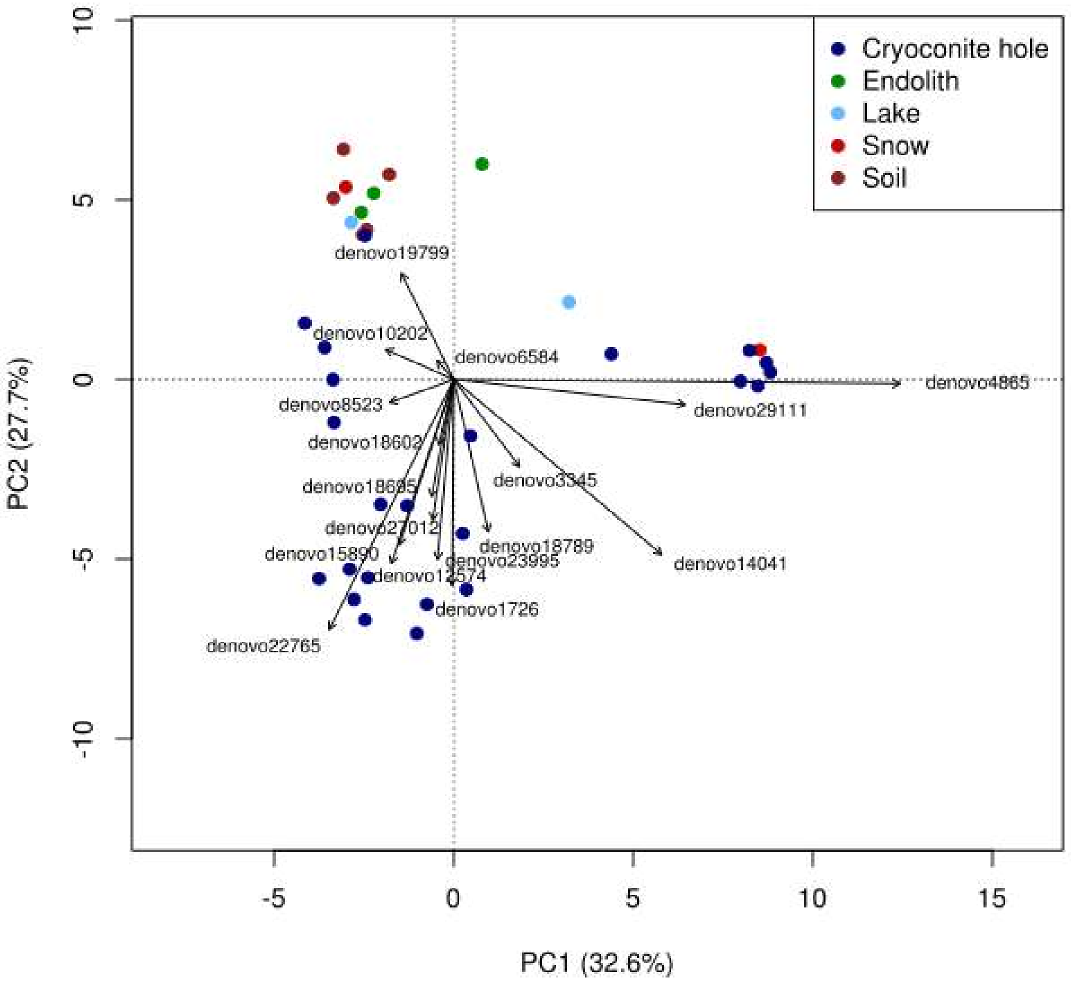

Figure S1. Principal component analysis of the bacterial OTUs. Samples cluster according to habitats.

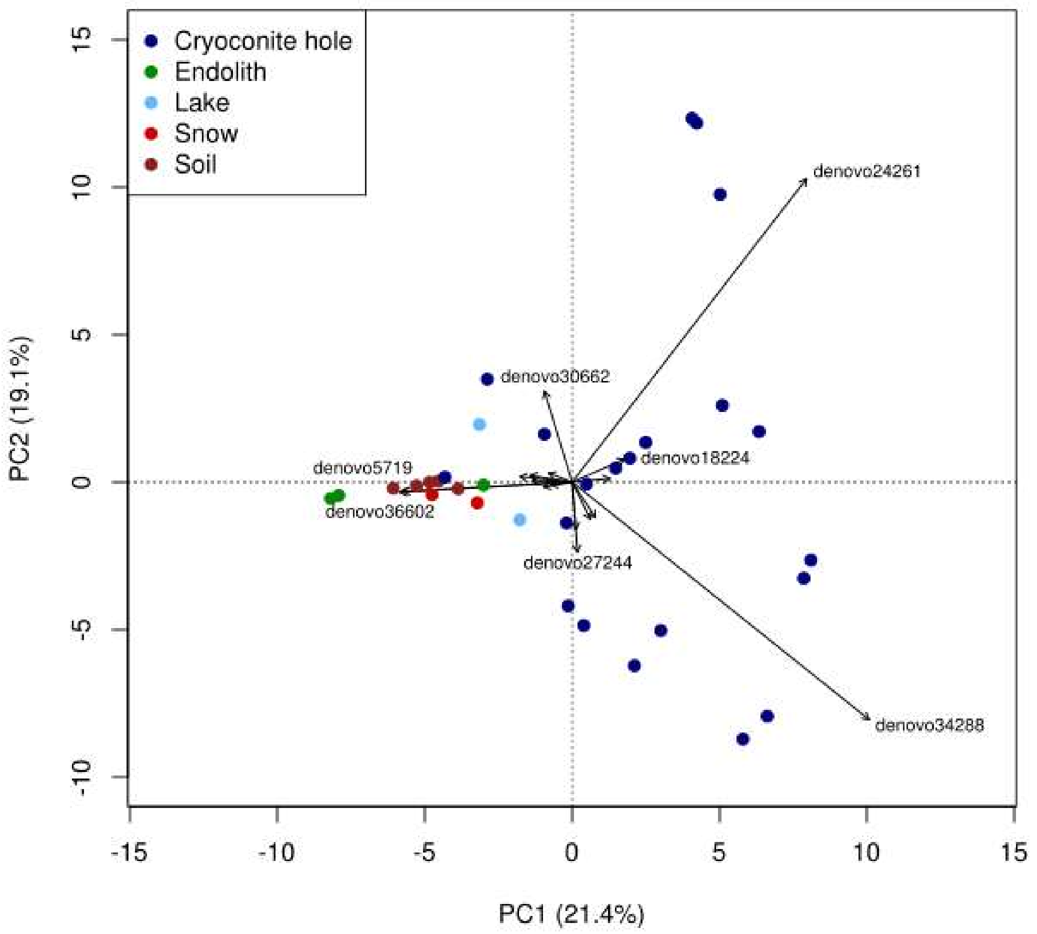

Figure S2. Principal component analysis of the eukaryotic OTUs. Samples cluster according to habitats.

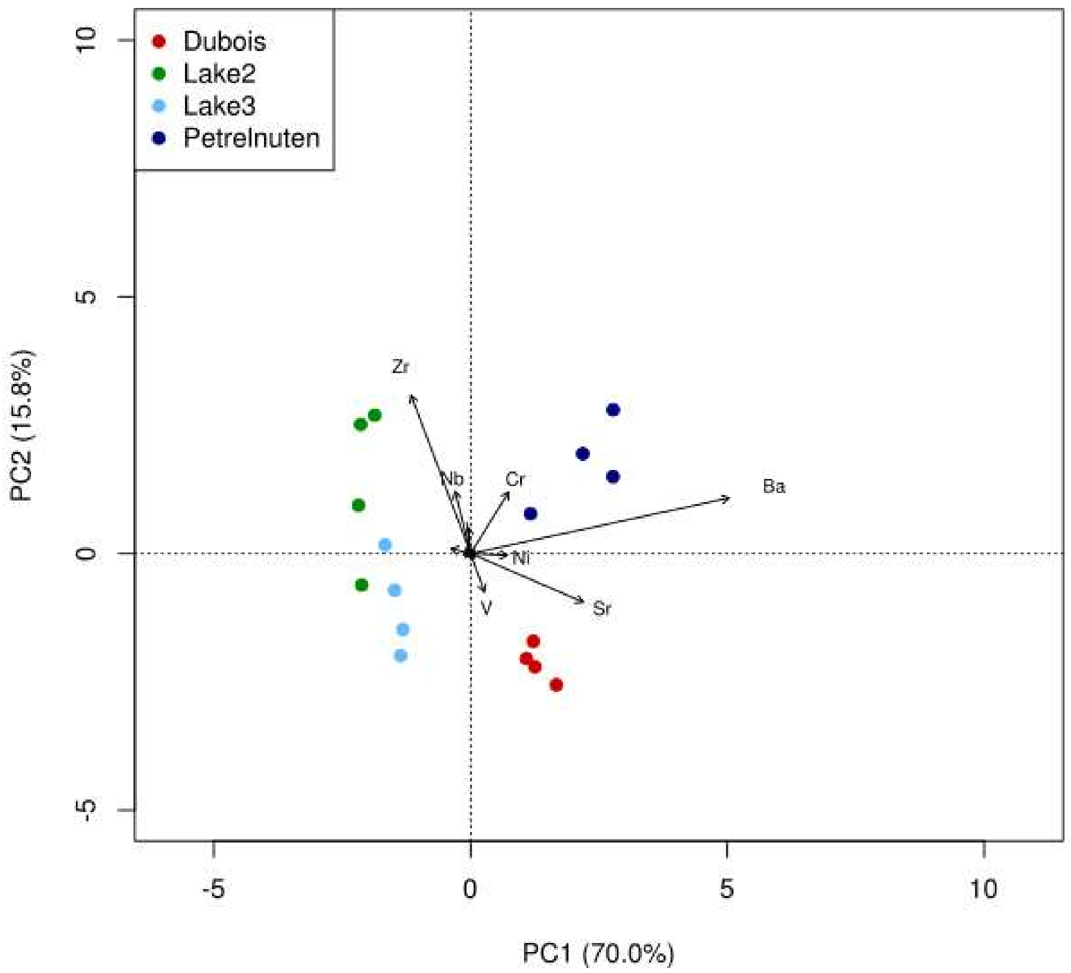

Figure S3. Principal component analysis of selected trace elements that cause the separation (primarily Zr, Ba and Sr).

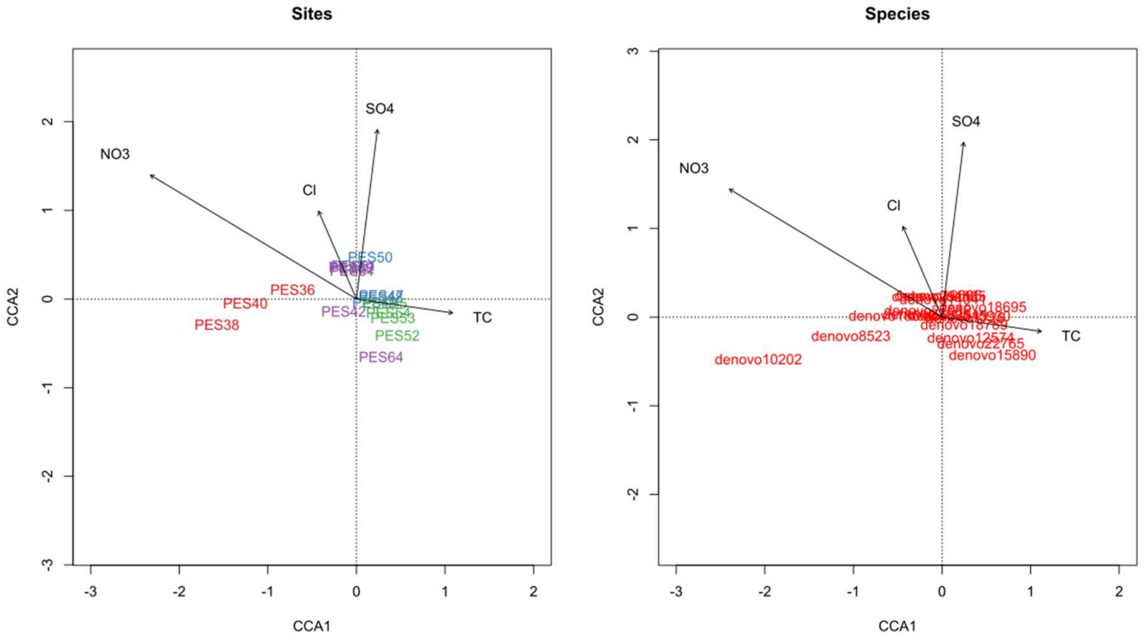

Figure S4. Partial canonical correspondence analysis of the bacterial abundances and all non-collinear environmental variables (i.e., after identification and exclusion of redundant predictor variables) and

without spatial effects. Samples from Lake 3 in Utsteinen clustered with higher nitrate concentration and samples from Dubois with a higher TC abundance. Otherwise no clear trends could be observed.

Table S1. Number of sequences before and after quality control for bacterial and eukaryotic sequences, respectively.

	**16S**		**18S**	
**Sample ID**	**Before quality filtering**	**After quality filtering**	**Before quality filtering**	**After quality filtering**
**PES17_36**	79285	71418	112519	112201
**PES17_38**	115832	111434	44238	44166
**PES17_39**	128336	123761	31865	31789
**PES17_40**	107580	104609	27128	27074
**PES17_42**	225182	218495	103515	103323
**PES17_43**	219156	213095	67378	67199
**PES17_47**	82531	79949	60130	59998
**PES17_48**	123666	120275	64459	64306
**PES17_49**	163446	158674	126366	126115
**PES17_50**	107304	104667	158362	158063
**PES17_51**	95033	93296	-	-
**PES17_52**	113682	110463	119486	119205
**PES17_53**	126238	122760	72656	72461
**PES17_54**	120805	117807	181725	181281
**PES17_55**	112134	108809	146821	146408
**PES17_56**	193142	187986	154063	153724
**PES17_59**	226518	220298	32560	32444
**PES17_60**	186567	182136	213031	212325
**PES17_61**	143702	140104	155784	155222
**PES17_62**	104661	102291	-	-
**PES17_63**	114068	111261	101205	100998
**PES17_64**	101054	98423	70930	70674
**PES17_65**	117504	113810	192746	192282
**Total**	3107426	3015821	2236967	2231258

Table S2. Distribution of 97% clustered bacterial OTUs assigned to the lowest possible taxonomic level. Values are the relative abundance of the taxa in percentage of total sequences and presented are all OTUs with >0.1% abundance.

Phylum	Class	Order; Family; Genus; Species	PES36	PES38	PES39	PES40	PES42	PES43	PES47	PES48	PES49	PES50	PES51	PES52
Acidobacteria	[Chloracido-bacteria]	RB41; Ellin6075	2.5	0.1	0	0	0.6	1.2	3.7	7.7	0.9	0.3	0.3	17.3
Actinobacteria	Acidimicrobiia	Acidimicrobiales; C111	0	<0.05	0	0	7.4	3.1	6.1	3.2	5.9	3.8	3.3	8.5
		Actinomycetales	1.1	1.0	0.2	1.3	4.5	1.4	12.9	9.7	4.4	7.4	4.4	7.9
		Actinomycetales; Geodermatophilaceae	0.5	0.7	1.2	3.1	2.3	0.6	1.9	2.3	1.1	6.2	3.6	0.6
		Actinomycetales; Intrasporangiaceae	0	0	0	0	0	0	0	0	0.2	6.0	4.0	0
		Actinomycetales; Kineosporiaceae	0	3.9	2.8	1.3	0	0	0	0	0	0	0	0
		Actinomycetales; Microbacteriaceae	2.4	0.1	5.5	0.1	0.1	0.1	0.0	0.0	0.1	0.1	0.1	0.1
		Actinomycetales; Microbacteriaceae; Yonghaparkia	1.8	4.0	1.0	4.2	9.5	12.3	3.9	6.4	5.9	22.5	14.0	5.8
		Actinomycetales; Nocardioidaceae	4.5	17.7	17.4	27.9	7.2	2.9	2.1	3.7	3.7	4.4	10.6	5.1
		Actinomycetales; Nocardioidaceae; Aeromicrobium	0.5	0.2	0.9	0.9	0.2	0.1	<0.05	<0.05	0.2	0.2	0.3	0.0
		Actinomycetales; Sporichthyaceae	1.2	17.3	9.5	17.4	8.6	1.5	7.7	6.9	9.2	13.6	17.4	6.4
	Thermoleo-philia	Gaiellales; AK1AB1_02E	0	0	<0.05	0	1.1	0.9	0.2	0.2	0.1	0.1	1.1	0.3
		Solirubrobacterales; Patulibacteraceae	0.4	0.9	0.8	2.5	0	0	0	<0.05	0	0	<0.05	0.1
Bacteriod etes	Cytopha gia	Cytophagales	0	0	0	0	0.1	3.3	0	0	0	0	0	0.1
		Cytophagales; Cyclobacteriaceae	0	0	0	0	<0.05	0.4	3.1	3.9	2.7	0.5	0.3	2.1

		Cytophagales; Cytophagaceae; Hymenobacter	2.7	25.2	25.1	12.6	0.5	0.5	0.9	5.3	4.3	1.4	0.5	0.6
		Cytophagales; Cytophagaceae; Spirosoma	0.3	0	0	<0.05	6.6	4.8	0.5	0.9	0.8	0.1	0.3	0.3
	Flavo- bacteriia	Flavobacteriales; Flavobacteriaceae; Flavobacterium	0.5	0	0	0	0.5	5.3	0	0	0	6.7	1.4	0
	Sphingo- bacteriia	Sphingobacteriales; Sphingobacteriaceae	0	0	0	0.0	0.0	0	0.1	1.1	0.0	0.0	0.1	0
	[Rhodo- thermi]	[Rhodothermales]; Rhodothermaceae; Rubricoccus	0.1	<0.05	0	0.0	1.7	1.5	0.0	0.3	<0.05	0.4	0.2	0
		[Saprospirales]; Chitinophagaceae	1.0	<0.05	0	0	6.9	3.1	0	0	0.7	0.1	1.0	0.8
		[Saprospirales]; Chitinophagaceae; Segetibacter	0	0	0	0	1.9	1.3	0	0	0.3	0.1	0.4	0.1
Chloroflexi	Thermo- microbia	JG30-KF-CM45	0	0	0	0	0.1	0.9	<0.05	<0.05	0	0.1	0.1	1.1
Cyanobacteria	Oscillatorio- phycideae	Chroococcales; Xenococcaceae	2.5	<0.05	<0.05	0.1	4.1	21.1	1.0	0.2	0.0	2.6	0.8	0.5
		Oscillatoriales; Phormidiaceae; Phormidium	1.4	0.1	<0.05	0	0.4	0	4.1	9.8	17.4	1.2	0.5	9.4
	Synechococco- phycideae	Pseudanabaenales; Pseudanabaenaceae	0.6	0	<0.05	0	0	0	<0.05	<0.05	0	4.5	1.3	0.8
FBP			0.5	<0.05	<0.05	0.1	4.1	9.0	0.5	0.7	0.8	0.1	0.3	0.1

Proteobacteria	Alphaproteobacteria	Caulobacterales; Caulobacteraceae; Mycoplana	0	0	0	0	<0.05	0.1	2.4	0.5	0.6	0.2	0.1	0.1
		Rhodobacterales; Rhodobacteraceae; Rubellimicrobium	0	0	0	0	4.8	1.9	3.3	2.9	2.3	2.6	1.0	0.9
		Rhodospirillales; Acetobacteraceae	0.1	0	0.1	0.7	5.0	1.3	0.1	0.1	0.5	1.0	0.6	0.2
		Rhodospirillales; Acetobacteraceae; Roseococcus	0.5	0	0	0	0.1	0.7	3.0	3.1	0.9	1.5	0.5	0.9
		Sphingomonadales; Sphingomonadaceae	0	<0.05	0.1	0	0.2	0.2	0	0	<0.05	0.1	0.1	0.1
		Sphingomonadales; Sphingomonadaceae; Kaistobacter	7.9	7.4	14.6	15.2	16.1	3.9	19.1	20.6	10.6	4.7	16.3	0.7
	Betaproteobacteria	Burkholderiales; Comamonadaceae	5.5	0.1	1.2	0.5	0.6	1.2	0.6	1.2	0.9	0.2	0.6	0.6
		Burkholderiales; Comamonadaceae	3.6	15.2	15.0	11.1	1.4	0.9	0.3	0.4	1.4	0.3	1.8	0.5
		Burkholderiales; Comamonadaceae; Hydrogenophaga	0.1	0	0	<0.05	<0.05	0.1	0.0	0.2	<0.05	2.5	0.6	<0.05
		Burkholderiales; Oxalobacteraceae	0.0	4.0	0.4	0.6	1.2	0	0	0	0	0	0	0
		Burkholderiales; Oxalobacteraceae; Janthinobacterium	1.0	<0.05	0	0	<0.05	0	0	0	0	0	0.1	0
	Gamma-proteo- bacteria	Xanthomonadales; Xanthomonadaceae	0.4	<0.05	0	0	0.7	5.6	0.1	0	<0.05	0.1	0.1	0.4
		Xanthomonadales; Xanthomonadaceae; Lysobacter	0	0	0	0	0.3	0.6	1.6	1.3	1.3	0.5	0.7	0.8
		Xanthomonadales; Xanthomonadaceae; Thermomonas	<0.05	0	0	0	0.1	1.0	15.3	2.6	17.8	3.2	5.0	17.2

Verrucomicrobia	Verrucomicrobiae	Verrucomicrobiales; Verrucomicrobiaceae	0.6	<0.05	<0.05	<0.05	1.1	5.6	4.0	3.1	3.9	0.6	0.7	2.3
		Verrucomicrobiales; Verrucomicrobiaceae	0.5	0	0	0	0	0	0.9	1.0	0.6	0.1	0.0	3.7
		[Chthoniobacterales]; [Chthoniobacteraceae]; DA101	0	<0.05	0	0	0	0	0.8	0.5	0.6	0.1	0.1	3.8
**Unassigned**			55.4	3.0	4.4	0.4	0.2	1.8	0.2	0.1	0.1	0.1	5.5	<0.05

Table S2. continued.

Phylum	Class	Order; Family; Genus; Species	PES53	PES54	PES55	PES56	PES59	PES60	PES61	PES62	PES63	PES64	PES65
**Acidobacteria**	**[Chloracido-bacteria]**	RB41; Ellin6075	12.4	11.2	7.1	1.6	0	0	0	0	<0.05	<0.05	3.5
**Actinobacteria**	**Acidimicrobiia**	Acidimicrobiales; C111	3.7	3.8	1.6	2.5	1.1	1.0	2.0	1.0	2.6	2.6	0.4
		Actinomycetales	6.2	11.5	13.3	9.3	0.3	0.6	1.4	2.3	1.7	1.4	0.2
		Actinomycetales; Geodermatophilaceae	0.6	0.9	1.6	1.5	0.2	0.3	0.3	0.3	1.1	0.2	0.7
		Actinomycetales; Intrasporangiaceae	0	0	0	0	0	0	0	0	0	0	0
		Actinomycetales; Kineosporiaceae	0	0	0	0	0	0	0	0	0	0	0
		Actinomycetales; Microbacteriaceae	0.1	<0.05	<0.05	0.1	0.5	0.4	0.2	0.3	0.3	0.1	1.7
		Actinomycetales; Microbacteriaceae; Yonghaparkia	5.2	7.1	6.6	8.7	18.9	19.7	19.3	16.7	18.7	16.0	5.7
		Actinomycetales; Nocardioidaceae	3.9	4.3	2.9	2.1	3.7	3.2	2.4	2.4	2.6	1.6	1.9
		Actinomycetales; Nocardioidaceae; Aeromicrobium	0.2	0.0	0.2	0.0	0.0	0	0	0	<0.05	0	1.2
		Actinomycetales; Sporichthyaceae	7.3	8.8	12.1	8.0	2.2	2.6	3.2	2.9	2.3	0.9	1.2
	**Th er**	Gaiellales; AK1AB1_02E	0.5	0.3	0.2	0.1	0	0	0	0	0	0	0.1

		Solirubrobacterales; Patulibacteraceae	0.2	0.1	0.1	0.0	0	0	0	0	0	0	<0.05
**Bacteriodetes**	**Cytophagia**	Cytophagales	0.1	0.2	0.1	<0.05	0	0	0	0	0	0	0
		Cytophagales; Cyclobacteriaceae	0.2	1.0	<0.05	0.9	0	0	0	0	0.0	1.2	0
		Cytophagales; Cytophagaceae; Hymenobacter	2.0	2.4	2.0	5.3	9.1	6.1	5.0	4.9	4.9	2.9	5.4
		Cytophagales; Cytophagaceae; Spirosoma	2.4	0.8	1.0	2.2	0.1	0.1	0.2	0.1	0.6	0	0.2
	**Flavo-bacterii**	Flavobacteriales; Flavobacteriaceae; Flavobacterium	0	0	0	0	0	0	0	0	0	15.3	0.6
	**Sphingo-bacteria**	Sphingobacteriales; Sphingobacteriaceae	0	0	0	0	0	0	0	0	0	3.1	0
	**[Rhodo-thermi]**	[Rhodothermales]; Rhodothermaceae; Rubricoccus	<0.05	<0.05	0.1	0.1	0	0	0	0	0.1	0.3	0.8
		[Saprospirales]; Chitinophagaceae	0.6	0.8	2.9	5.1	<0.05	<0.05	0	0	0	0	1.0
		[Saprospirales]; Chitinophagaceae; Segetibacter	0.1	0.2	0.3	<0.05	0	0	0	0	0	0	1.8
**Chloroflexi**	**Thermo-microbia**	JG30-KF-CM45	0.1	0.4	0.1	0.1	0	0	0	0	0	0.1	0.4
**Cyanobacteria**	**Oscillatorio-phycideae**	Chroococcales; Xenococcaceae	1.7	4.7	6.5	7.0	45.9	48.2	40.9	52.8	43.2	0.3	0.3
		Oscillatoriales; Phormidiaceae; Phormidium	18.9	20.2	20.0	15.0	0	0	0	0	<0.05	27.8	38.0

	Synechococco-phycideae	Pseudanabaenales; Pseudanabaenaceae	12.0	4.4	6.9	12.3	0	0	0	0	0	0	0
FBP			0.2	0.1	0.1	0.1	<0.05	<0.05	<0.05	<0.05	0.1	2.0	<0.05
Proteobacteria	Alphaproteobacteria	Caulobacterales; Caulobacteraceae; Mycoplana	0	<0.05	0	0.1	0	0	0	0	0.1	3.8	3.8
		Rhodobacterales; Rhodobacteraceae; Rubellimicrobium	0.9	1.1	0.6	1.2	0.3	0.3	0.5	0.1	0.9	0.7	0.5
		Rhodospirillales; Acetobacteraceae	0.6	0.3	0.3	0.5	0.1	0.1	0.1	<0.05	0.6	0.2	0.2
		Rhodospirillales; Acetobacteraceae; Roseococcus	0.5	1.1	0.3	1.7	0.0	<0.05	0.0	<0.05	1.2	2.9	0.5
		Sphingomonadales; Sphingomonadaceae	0.3	0.1	0.1	0.1	0	0	<0.05	0	0.0	<0.05	8.9
		Sphingomonadales; Sphingomonadaceae; Kaistobacter	5.5	4.1	3.1	6.0	2.7	2.3	5.3	3.0	3.9	2.4	1.8
	Betaproteo- bacteria	Burkholderiales; Comamonadaceae	0.7	0.5	0.3	0.5	0.1	0.1	0.3	0.2	1.2	0.2	1.2
		Burkholderiales; Comamonadaceae	0.7	0.8	1.8	0.3	0.1	<0.05	<0.05	<0.05	0.1	<0.05	1.3
		Burkholderiales; Comamonadaceae; Hydrogenophaga	0	0	0	0	<0.05	<0.05	0.1	0.1	0.4	3.9	1.3
		Burkholderiales; Oxalobacteraceae	0	0	0	0	0	0	0	0	0	0	0
		Burkholderiales; Oxalobacteraceae; Janthinobacterium	0	0	0	0	0	0	0	0	0	0	4.8
	Gamma-proteobacteria	Xanthomonadales; Xanthomonadaceae	0.3	0.7	0.7	1.7	10.8	12.2	17.1	11.1	11.9	1.0	0.2
		Xanthomonadales; Xanthomonadaceae; Lysobacter	2.3	0.8	0.5	0.3	1.6	1.5	0.7	0.2	0.3	0.6	0.2
		Xanthomonadales; Xanthomonadaceae; Thermomonas	4.4	3.2	1.1	2.1	0	0	0	0	0.0	8.0	0
Verrucomicrobia	Verruco-	Verrucomicrobiales; Verrucomicrobiaceae	1.2	1.2	0.6	0.2	<0.05	<0.05	<0.05	<0.05	<0.05	0.1	0.4

		Verrucomicrobiales; Verrucomicrobiaceae	1.4	1.4	0.3	2.1	0	0	0	0	0	0.3	4.3
		[Chthoniobacterales]; [Chthoniobacteraceae]; DA101	2.9	1.6	1.0	1.4	0	0	0	0	0	0.1	4.8
**Unassigned**			<0.05	0.1	3.7	0.0	2.2	1.3	1.1	1.7	1.1	0.2	0.8

Table S3. The core bacterial taxa present in all cryoconite hole samples at >1% relative abundance.

Phylum	Class	Order	Family; Genus; Species	OTU ID	PES36	PES38	PES39	PES40	PES42	PES43	PES47	PES48	PES49	PES50	PES51	PES52
**Actinobacteria**	Actinobacteria	Actinomycetales		denovo1726	0.2	<0.05	0.2	1.1	4.1	1.3	12.0	8.8	4.0	6.8	3.6	7.2
			Geodermatophilaceae	denovo19685	0.1	0.6	1.0	2.7	2.1	0.5	1.7	2.1	1.0	5.7	2.9	0.5
			Microbacteriaceae; Yonghaparkia	denovo14041	0.3	3.5	0.9	3.7	8.7	11.3	3.6	5.8	5.4	20.7	11.2	5.3
			Nocardioidaceae	denovo18602	0.7	14.5	12.6	17.4	3.5	1.4	0.8	1.4	1.2	1.8	4.4	0.6
			Sporichthyaceae	denovo23995	0.2	15.0	8.3	15.4	7.9	1.4	7.2	6.3	8.5	12.6	13.9	5.9
			Nocardioidaceae	denovo15045	0	<0.05	0.1	0.5	1.0	0.3	0.1	0.2	0.3	0.3	1.0	3.2
			Nocardioidaceae	denovo22480	0	<0.05	0.1	0.9	1.4	0.4	0.2	0.3	0.4	0.4	1.7	0.2
			Sporichthyaceae	denovo3326	<0.05	<0.05	<0.05	<0.05	0.2	<0.05	0.1	0.1	0.1	0.2	<0.05	0.1
**Cyanobacteria**	Oscillatoriophycideae	Chroococcales	Xenococcaceae	denovo4865	0.5	<0.05	<0.05	<0.05	3.7	19.1	0.9	0.1	<0.05	2.4	0.7	0.5
**FBP**				denovo15099	<0.05	<0.05	<0.05	0.1	3.2	5.1	<0.05	<0.05	<0.05	<0.05	0.3	0.1
**Proteobacteria**	Alphaproteobacteria	Sphingomonadales	Sphingomonadaceae; Kaistobacter	denovo27012	1.5	6.4	12.7	13.4	10.8	2.7	14.7	15.1	9.0	3.9	12.9	0.5
		Rhodospirillales	Acetobacteraceae	denovo19929	<0.05	<0.05	0	0.6	4.6	1.2	0.1	0.1	0.4	0.9	0.5	0.2

	Betaproteo- bacteria	Burkholderiales	Xenococcaceae	denovo6584	1.1	0.1	1.0	0.4	0.6	1.1	0.6	1.1	0.9	0.2	0.5	0.5

Table S3. continued.

Phylum	Class	Order	Family; Genus; Species	OTU ID	PES53	PES54	PES55	PES56	PES59	PES60	PES61	PES62	PES63	PES64	PES65
**Actinobacteria**	Actinobacteria	Actinomycetales		denovo1726	5.8	10.9	12.2	8.7	0.3	0.5	1.3	2.2	1.5	1.3	0.2
			Geodermatophilaceae	denovo19685	0.6	0.9	1.5	1.4	0.2	0.2	0.2	0.3	1.1	0.2	0.6
			Microbacteriaceae; Yonghaparkia	denovo14041	4.8	6.8	6.0	8.1	17.8	19.0	18.6	16.1	17.5	14.6	4.8
			Nocardioidaceae	denovo18602	1.2	1.3	1.1	0.7	1.0	1.0	0.8	0.9	0.9	0.7	0.9
			Sporichthyaceae	denovo23995	6.8	8.4	11.1	7.4	2.1	2.5	3.0	2.8	2.2	0.8	1.0
			Nocardioidaceae	denovo15045	1.7	1.9	0.8	0.5	<0.05	<0.05	<0.05	<0.05	0.1	0.1	0.2
			Nocardioidaceae	denovo22480	0.5	0.4	0.4	0.2	<0.05	<0.05	<0.05	<0.05	0.1	0.2	0.3
			Sporichthyaceae	denovo3326	0.1	<0.05	<0.05	0.2	0.2	0.1	0.1	0.1	0.1	<0.05	<0.05
**Cyanobacteria**	Oscillatoriop- phycideae	Chroococcales	Xenococcaceae	denovo4865	1.6	4.5	5.9	6.4	42.6	46.0	39.1	50.7	39.8	0.2	0.3
**FBP**				denovo15099	0.1	0.1	0.1	<0.05	<0.05	<0.05	<0.05	<0.05	0.1	<0.05	<0.05
**Proteobacteria**	Alphaproteobacteria	Sphingo- monadales	Sphingomonadaceae; Kaistobacter	denovo27012	3.9	3.2	2.4	4.2	1.8	1.6	3.7	2.0	2.4	1.5	1.4
		Rhodo- spirillales	Acetobacteraceae	denovo19929	0.5	0.3	0.2	0.4	0.1	0.1	0.1	<0.05	0.6	0.2	0.2
	Betaproteo- bacteria	Burkholderiales	Xenococcaceae	denovo6584	0.6	0.5	0.3	0.4	0.1	0.1	0.3	0.2	1.1	0.2	1.0

Table S3. continued: continued. The NCBI hits of core bacterial taxa.

Phylum	Class	Order	Family; Genus; Species	OTU ID	Best NCBI hit	Accession number	Similarity [%]
**Actinobacteria**	Actinobacteria	Actinomycetales		denovo1726	Uncultured actinobacterium	FJ490312.1	99
			Geodermatophilaceae	denovo19685	Blastococcus sp.	EU704838.1	99
			Microbacteriaceae; Yonghaparkia	denovo14041	Leifsonia sp.	KT965169.1	99
			Nocardioidaceae	denovo18602	Nocardioides sp.	KY386518.1	99
			Sporichthyaceae	denovo23995	Uncultured actinobacterium	EU931108.1	99
			Nocardioidaceae	denovo15045	Nocardioides sp.	GQ420967.1	99
			Nocardioidaceae	denovo22480	Nocardioides sp.	GQ420967.1	97
			Sporichthyaceae	denovo3326	Leifsonia sp.	KT965169.1	96
**Cyanobacteria**	Oscillatorio-phycideae	Chroococcales	Xenococcaceae	denovo4865	Uncultured cyanbacterium	HQ230210.1	99
**FBP**				denovo15099	Uncultutred endolithic bacterium	AB374370.1	99
**Proteobacteria**	Alphaproteobacteria	Sphingo-monadales	Sphingomonadaceae; Kaistobacter	denovo27012	Sphingomonas sp.	MF002313.1	99
		Rhodo-spirillales	Acetobacteraceae	denovo19929	Uncultured bacterium	FR667548.1	97
	Betaproteo-bacteria	Burkholderiales	Comamonadaceae	denovo6584	Polaromonas sp.	KF414342.1	99

Table S4. Taxonomic identification of the discriminating OTUs in the principal component analysis (Figure 3).

OTU ID	Phylum	Class	Order	Family	Genus	Species	Best NCBI hit	Accession number	Similarity [%]
**denovo1726**	Actinobacteria	Actinobacteria	Actinomycetales	-	-	-	Uncultured actinobacterium	FJ490312.1	99
**denovo3345**	Bacteriodetes	Cytophagia	Cytophagales	Cytophagaceae	*Hymenobacter*	-	Uncultured Bacteriodetes	KC442558.1	98
**denovo4865**	Cyanobacteria	Oscillatoriophycideae	Chroococcales	Xenococcaceae	-	-	Uncultured cyanbacterium	HQ230210.1	99
**denovo6584**	Proteobacteria	Betaproteobacteria	Burkholderiales	Comamonadaceae	-	-	Polaromonas sp.	KF414342.1	99
**denovo8523**	Proteobacteria	Betaproteobacteria	Burkholderiales	Comamonadaceae	-	-	Uncultured Comamonadaceae	GQ454852.1	99
**denovo10202**	Bacteriodetes	Cytophagia	Cytophagales	Cytophagaceae	*Hymenobacter*	-	Hymenobacter sp. R-36490	FR682723.1	99
**denovo12574**	Proteobacteria	Gammaproteobacteria	Xanthomonadales	Xanthomonadaceae	*Thermomonas*	-	Uncultured Gammaproteobacterium	FR749755.1	99
**denovo14041**	Actinobacteria	Actinobacteria	Actinomycetales	Microbacteriaceae	*Yonghaparkia*	-	Leifsonia sp.	KT965169.1	99
**denovo15890**	Acidobacteria	[Chloracidobacteria]	RB41	Ellin6075	-	-	Uncultured Blastocatella sp.	LT625222.1	99
**denovo15930**	Proteobacteria	Alphaproteobacteria	Rhodobacterales	Rhodobacteraceae	*Rubellimicrobium*	-	Rubellimicrobium sp	JQ401336.1	99
**denovo18602**	Actinobacteria	Actinobacteria	Actinomycetales	Nocardioidaceae	-	-	Nocardioides sp.	KY386518.1	99
**denovo18695**	Cyanobacteria	Synechococcophycideae	Pseudanabaenales	Pseudanabaenaceae	-	-	Leptolyngbya sp. LEGE 07075	HM217080.1	98
**denovo18789**	Actinobacteria	Acidimicrobiia	Acidimicrobiales	C111	-	-	Uncultured Actinobacterium	EF628488.1	99
**denovo19685**	Actinobacteria	Actinobacteria	Actinomycetales	Geodermatophilaceae	-	-	Blastococcus sp.	EU704838.1	99
**denovo19799**	Acidobacteria	[Chloracidobacteria]	RB41	Ellin6075	-	-	Uncultured acidobacterium	JQ402009.1	99
**denovo22765**	Cyanobacteria	Oscillatoriophycideae	Oscillatoriales	Phormidiaceae	*Phormidium*	-	Phormidium sp. CCALA 846	GQ504034.1	99
**denovo23995**	Actinobacteria	Actinobacteria	Actinomycetales	Sporichthyaceae	-	-	Agrococcus sp.	KF029486.1	99

Table S5. Distribution of 99% clustered eukaryotic OTUs assigned to the lowest possible taxonomic level. Values are the relative abundance of the taxa in percentage of total sequences and presented are all OTUs with >0.1% abundance.

				PES36	PES38	PES39	PES40	PES42	PES43	PES47	PES48	PES49	PES50	PES52
**Archaeplastida**	Chloroplastida	Charophyta	Brassicales; Brassica; Brassica oleracea var. capitata	0.6	<0.05	0	0	0	0	0	0	0	0	0
			Poales; Triticum; Triticum aestivum	0.6	0	0	0	0	<0.05	0	<0.05	0	0	<0.05
			Phragmoplastophyta; Zygnematophyceae; Mesotaenium sp. AG-2009-1	4.4	0.1	0.1	0	0	0	<0.05	<0.05	<0.05	<0.05	0
		Chlorophyta	Chlorophyceae; Chlorococcum oleofaciens; Chlorococcum oleofaciens	0.2	0	<0.05	0	<0.05	0.2	0	0	0	<0.05	78.8
			Trebouxiophyceae; Actinastrum hantzschii; Actinastrum hantzschii	1.9	<0.05	95.3	96.3	0.1	0.5	18.1	89.4	1.0	21.9	7.0
			Trebouxiophyceae; uncultured Dunaliellaceae	30.1	0.2	0	0.2	0	0	0	0	0	0	0
			Trebouxiophyceae; uncultured eukaryote	4.7	0.1	0.0	0	0	<0.05	0.0	0.2	0	0	0
			Chlorodendrales; Tetraselmis; Tetraselmis subcordiformis	0	0	0	0	0.0	0	0	0	0	0	0.0
			Prasiolales; Prasiola; Prasiola furfuracea	37.6	0.2	0.1	0.0	0.1	0.0	<0.05	<0.05	0	0	0
			Prasiolales; Stichococcus; Stichococcus jenerensis	1.4	98.8	4.3	3.4	48.6	0.2	<0.05	<0.05	0.0	0.1	<0.05
	Rhodopycea		Bangiales; Pyropia; Pyropia fallax	1.3	<0.05	0.0	0.1	1.5	26.2	80.7	2.3	95.4	77.9	2.3
**Excavata**	Discoba	Discicristat	Tetramitia; AND12; Heterolobosea sp. OSA	0	0	0	0	0	<0.05	0	0.1	0	0	0
**Opisthokonta**	Holozoa	Metazoa	Bdelloidea; Adinetida; Hypsibius dujardini	3.7	0.1	0	0	0	0	0	0	0	0	<0.05
			Eutardigrada; Parachela; uncultured eukaryote	2.6	0.4	0	0	0	0	0	0.1	0	0	0

	Nucletmycea	Fungi	Eurotiomycetes; Eurotiales; Penicillium sp. NS051-06	0.8	0	0	0	0	0	0	0	0	0	0
			Eurotiomycetes; Eurotiales; Talaromyces radicus	0	0	0	0	0	0	0	0	0	0	0
			Saccharomycetes; Saccharomycetales; Saccharomyces cerevisiae Kyokai no. 7	0	0	0	0	0	<0.05	<0.05	<0.05	0	0	0
			Incertae Sedis; Hyaloraphidium; Hyaloraphidium curvatum	0	0	0	0	<0.05	0	0.1	0.2	0.1	0	0
			Incertae Sedis; Malassezia; uncultured eukaryote	4.0	0.1	0.1	0	<0.05	<0.05	0	0	0	0	<0.05
			Incertae Sedis; Malassezia; uncultured fungus	0	0	0	0	0	0	0	0.0	0	0	0
**SAR**	Alveolata	Ciliophora	Phyllopharyngea; Cyrtophoria; Colepidae environmental sample	0	0	0	0	0	0	0	0	0	0	0
	Rhizaria	Cercozoa	Glissomonadida; Heteromita; Cercozoa sp. B128	0.3	<0.05	0	0	29.1	<0.05	<0.05	0	0	0	0
			Glissomonadida; Heteromita; uncultured cercozoan	0	0	0	0	0.1	12.2	0	0	0.4	0	0
			Glissomonadida; Heteromita; uncultured eukaryote	0.5	0	0	0	0	0	0	0	0	<0.05	11.6
			Glissomonadida; uncultured eukaryote	0	0	0	0	0.8	57.4	0	0	0	0	0
			Thecofilosea; E-A1	0	0	0	0	0	0	0	0.0	0	0	0
			Thecofilosea; uncultured eukaryote	0	0	0	<0.05	19.7	3.3	1.1	7.5	3.1	0.1	0.3
	Stramenopiles	Ochrophyta	Chrysophyceae; uncultured eukaryote	1.4	0	0	0	0	0	0	<0.05	0	0	0
			Chrysocapsales; Hydrurus; uncultured Hydrurus	4.0	<0.05	0	0	0	<0.05	0	0	0	<0.05	<0.05
**Unassigned**				0	0	0	0	<0.05	0.1	0	0	0	0	0

Table S5. continued.

				PES53	PES54	PES55	PES56	PES59	PES60	PES61	PES63	PES64	PES65
**Archaeplastida**	Chloroplastida	Charophyta	Brassicales; Brassica; Brassica oleracea var. capitata	0	0.1	0	<0.05	0.2	0.4	1.3	0	0	0
			Poales; Triticum; Triticum aestivum	<0.05	0.2	0	0	15.3	60.6	30.8	<0.05	<0.05	0
			Phragmoplastophyta; Zygnematophyceae; Mesotaenium sp. AG-2009-1	<0.05	1.2	<0.05	<0.05	0.1	0.6	0.7	<0.05	0	0
		Chlorophyta	Chlorophyceae; Chlorococcum oleofaciens; Chlorococcum oleofaciens	1.1	0.3	0	0	3.1	0.2	0.2	7.0	28.1	0.1
			Trebouxiophyceae; Actinastrum hantzschii; Actinastrum hantzschii	6.7	35.1	2.4	28.6	0.1	0.2	<0.05	0.2	<0.05	0.6
			Trebouxiophyceae; uncultured Dunaliellaceae	0	0.1	0	0	0	0.0	0	0	0	0
			Trebouxiophyceae; uncultured eukaryote	0	0.1	0	<0.05	0.2	0.1	0.3	0	0	0
			Chlorodendrales; Tetraselmis; Tetraselmis subcordiformis	0.2	2.1	0	<0.05	0	0	0	0	0	<0.05
			Prasiolales; Prasiola; Prasiola furfuracea	0	0.1	0	<0.05	22.8	3.3	2.9	0.7	0	0.1
			Prasiolales; Stichococcus; Stichococcus jenerensis	0	0	<0.05	<0.05	0.0	0.3	0.5	0	<0.05	<0.05
	Rhodopycea		Bangiales; Pyropia; Pyropia fallax	5.4	19.1	4.3	30.0	30.7	6.9	14.2	85.7	0.9	0.9
**Excavata**	Discoba	Discicristat	Tetramitia; AND12; Heterolobosea sp. OSA	0	0	0	0	0	0	0	0	8.1	0.0
**Opisthoko**	Holozoa	Metazoa	Bdelloidea; Adinetida; Hypsibius dujardini	<0.05	0.1	0	0	0	<0.05	0.1	0	0	0
			Eutardigrada; Parachela; uncultured eukaryote	0	0.1	0	0	0	0	0	0	0	0

	Nucletmycea	Fungi	Eurotiomycetes; Eurotiales; Penicillium sp. NS051-06	<0.05	<0.05	0	0	0.8	2.2	1.6	0	0	0
			Eurotiomycetes; Eurotiales; Talaromyces radicus	0	0	0	0	1.7	3.8	1.1	<0.05	0	0
			Saccharomycetes; Saccharomycetales; Saccharomyces cerevisiae Kyokai no. 7	0	0.1	0	0	5.2	13.3	17.9	<0.05	0	0
			Incertae Sedis; Hyaloraphidium; Hyaloraphidium curvatum	0	0	0	0	0	0	0.2	0.1	31.2	0.1
			Incertae Sedis; Malassezia; uncultured eukaryote	0.1	0.7	0	0	1.0	1.7	2.0	0	0	0
			Incertae Sedis; Malassezia; uncultured fungus	0	0.1	0	0	0.2	0.8	2.5	0	0	0
**SAR**	Alveolat	Ciliophor	Phyllopharyngea; Cyrtophoria; Colepidae environmental sample	0	0	0	0	0.0	0	0	0	2.5	29.5
	Rhizaria	Cercozoa	Glissomonadida; Heteromita; Cercozoa sp. B128	0	0	<0.05	0	0	<0.05	0	0	0	0.0
			Glissomonadida; Heteromita; uncultured cercozoan	0	0	0	0	0	<0.05	0.0	0	8.4	0.6
			Glissomonadida; Heteromita; uncultured eukaryote	4.1	26.1	<0.05	10.3	0	<0.05	0	0	0.0	0
			Glissomonadida; uncultured eukaryote	0	0.1	0	0.0	0	<0.05	0.1	0	0.2	26.1
			Thecofilosea; E-A1	22.0	0.0	<0.05	2.6	0	0	0	0	0	<0.05
			Thecofilosea; uncultured eukaryote	60.5	13.3	93.2	28.2	2.8	0.4	0.9	6.0	20.4	41.9
	Strameno-	Ochrophyta	Chrysophyceae; uncultured eukaryote	<0.05	0.3	0	<0.05	0	0.2	0.2	0.0	0	0
			Chrysocapsales; Hydrurus; uncultured Hydrurus	<0.05	0.9	0	<0.05	0.1	0.4	0.6	<0.05	<0.05	0
**Unassigned**				0	<0.05	0	0.1	15.8	4.6	22.0	0.3	0	0

Table S6. The core eukaryotic taxa present in all cryoconite hole samples at >1% relative abundance.

OTU ID	PES36	PES38	PES39	PES40	PES42	PES43	PES47	PES48	PES49	PES50	PES52	PES53	PES54	PES55	PES56	PES59	PES60	PES61	PES63	PES64	PES65
**denovo9728**	1.4	<0.05	94.5	95.5	0.1	0.5	17.9	87.3	1.0	21.7	6.9	6.0	32.8	2.3	27.5	0.1	0.1	<0.05	0.1	<0.05	0.6
**denovo13741**	1.0	<0.05	<0.05	0.1	1.5	25.8	79.8	2.3	94.0	77.3	2.2	4.9	17.8	4.2	28.9	25.7	4.9	10.0	85.0	0.9	0.8

Table S6. continued. The closest NCBI hits of core Eukaryotic taxa.

OTU ID						NCBI	Accession number	Similarity [%]
**denovo9728**	Archaeplastida	Chloroplastida	Chlorophyta	Trebouxiophyceae	Actinastrum hantzschi	Chlorella sp.		100
**denovo13741**		Rhodophceae	Bangiales	Pyropia	Pyropia fallax	Porpyrax fallax, Ulothrix zonata		99

Table S7. Taxonomic identification of the discriminating OTUs in the principal component analysis (Figure 5).

OTU ID	Unranked	Division	Class	Order	Family	Genus	Species	Best NCBI hit	Similarity [%]
**denovo5719**	Rhizaria	Cercozoa	Sarcomonadea	Glissomonadida	Heteromitidae	*Heteromita*		Unculutred eukaryote clone	99
**denovo7317**	Rhizaria	Cercozoa	Thecofilosea					Rhogostoma cylindrica	99
**denovo9728**	Archaeplastida	Chlorophyta	Trebouxiophyceae	Chlorellales	Chlorellaceae	*Actinastrum*	*Actinastrum hantzschi*	Chlorella sp.	100
**denovo10938**	Archaeplastida	Charophyta	Spermatophyta	Poales	Poaceae	*Triticum*	*Triticum aestivum*	Triticum aestivum, Uncultured fungal clone	100
**denovo12309**	Archaeplastida	Chlorophyta	Trebouxiophyceae	Prasiolales	Prasiolaceae	*Stichococcus*	*Stichococcus jenerensis*	Chlorella spp./Raphidonema spp./Stichococcus spp.	99
**denovo13741**	Archaeplastida	Rhodophyta	Bangiophyceae	Bangiales	Bangiaceae	*Pyropia*	*Pyropia fallax*	Porpyrax fallax, Ulothrix zonata	99
**denovo18224**	Rhizaria	Cercozoa	Thecofilosea					No blast hit	
**denovo24261**	Archaeplastida	Rhodophyta	Bangiophyceae	Bangiales	Bangiaceae	*Pyropia*	*Pyropia fallax*	Porpyrax fallax, Ulothrix zonata	99
**denovo27244**	Metazoa	Rotifera	Bdelloidea	Adinetida	Adinetidae	*Adineta*	*Adineta vaga*	Adineta vaga	99
**denovo30662**	Fungi	Ascomycota	Lecanoromycetes	Acarosporales	Acarosporaceae	*Acarospora*	*Acarospora peliscypha*	Acarospora spp.	99
**denovo34288**	Fungi	Ascomycota	Thelebolaceae	Thelebolales	Thelebolaceae	*Thelebolus*	*Thelebolus stercoreus*	Thelebolus spp.	99
**denovo30662**	Fungi	Ascomycota	Lecanoromycetes	Acarosporales	Acarosporaceae	*Acarospora*	*Acarospora peliscypha*	Acarospora spp.	99

**denovo29111**	Archaeplastida	Chlorophyta	Chlorophyceae	Chlorococcales	Chlorococcaceae	*Chlorococcum*	*Chlorococcum oleofaciens*	Chlorococcum spp./Pleurastrum spp./Macrochloris spp.	99

Table S8. Overview of solid total carbon (TC), total organic carbon (TOC), total nitrogen (N), total organic nitrogen (TON), thermogravimetry, thermovaporization and pyrolysis analyses.

Sample	Location	TC [%]	TOC [%]	TN [%]	TON [%]	Total mass loss in TGA [%]	TOC [%]	Thermovaporization (300°C) Live C [mg/g TOC]	Pyrolysis (300–600°C) Labile C [mg/g TOC]
**PES36**	Lake 3	0.12	0.02	0.01	<0.01	0.52	0.017	0	58
**PES37**	Lake 3	0.12	0.09	0.01	<0.01	-			
**PES38**	Lake 3	0.02	b.d.	<0.01	<0.01	0.78			
**PES39**	Lake 3	0.02	0.02	<0.01	<0.01	0.95			
**PES40**	Lake 3	0.02	0.03	<0.01	<0.01	0.63			
**PES47**	Lake 2	0.10	0.05	<0.01	<0.01	0.47			
**PES48**	Lake 2	0.22	0.05	0.01	<0.01	1.00			
**PES49**	Lake 2	0.13	0.10	<0.01	<0.01	1.02	0.095	0	55
**PES50**	Lake 2	0.04	0.08	<0.01	<0.01	0.21			
**PES51**	Lake 2	0.03	0.02	<0.01	<0.01	0.62			
**PES52**	Dubois	0.05	0.03	<0.01	<0.01	1.12			
**PES53**	Dubois	0.03	0.04	<0.01	<0.01	0.61			
**PES54**	Dubois	0.07	0.03	<0.01	<0.01	0.65			
**PES55**	Dubois	0.05	0.05	<0.01	<0.01	0.78	0.047	0	74
**PES56**	Dubois	0.03	0.04	<0.01	<0.01	0.42			
**PES42**	Petrelnuten	0.06	0.03	<0.01	<0.01	0.61			
**PES43**	Petrelnuten	0.11	0.08	<0.01	<0.01	-			
**PES59**	Petrelnuten	0.12	0.03	0.01	<0.01	0.35			
**PES60**	Petrelnuten	0.13	0.12	<0.01	<0.01	-			
**PES61**	Petrelnuten	0.08	0.12	<0.01	<0.01	0.33	0.123	0	17
**PES62**	Petrelnuten	0.10	0.14	<0.01	<0.01	0.88	0.136	0	5
**PES63**	Petrelnuten	0.24	0.10	0.01	<0.01	0.35	0.103	0	10
**PES64**	Petrelnuten	0.16	0.23	0.01	<0.01	0.96	0.229	0	19
**PES65**	Petrelnuten	0.14	0.12	0.01	<0.01	0.98			

Table S9. Chemical composition of cryoconite sediments (expressed as oxides) from X-ray fluorescence (XRF) analysis.

Sample	Location	SiO2 [%]	TiO2 [%]	Al2O3 [%]	Fe2O3 [%]	MnO [%]	MgO [%]	CaO [%]	Na2O [%]	K2O [%]	P2O5 [%]	LOI Tiegel [%]	Sum [%]
**PES36**	Lake 3	63.6	0.495	15.8	5.81	0.093	1.43	2.55	3.31	5.84	0.053	0.63	99.67
**PES38**	Lake 3	58.6	0.679	16.4	8.44	0.135	2.22	3.78	3.36	5.05	0.060	0.83	99.57
**PES39**	Lake 3	55.8	0.933	15.9	10.65	0.174	3.06	4.91	3.01	3.93	0.080	1.14	99.57
**PES40**	Lake 3	62.6	0.410	17.0	5.02	0.082	1.29	2.18	3.50	7.01	0.044	0.66	99.72
**PES47**	Lake 2	68.5	0.372	14.1	4.77	0.073	0.45	1.64	3.24	5.99	0.053	0.49	99.61
**PES48**	Lake 2	72.2	0.224	13.2	3.00	0.044	0.18	0.94	3.13	5.75	0.028	1.01	99.66
**PES49**	Lake 2	65.7	0.435	15.0	5.30	0.082	0.77	1.91	3.56	5.81	0.071	0.99	99.63
**PES50**	Lake 2	73.4	0.150	12.9	2.33	0.034	0.05	0.64	2.83	6.18	0.014	1.25	99.82
**PES52**	Dubois	65.9	0.529	15.0	4.90	0.088	1.94	3.63	3.56	3.04	0.143	0.90	99.65
**PES53**	Dubois	67.5	0.310	16.1	3.09	0.059	1.12	2.90	3.95	3.86	0.080	0.71	99.62
**PES54**	Dubois	68.3	0.355	15.1	3.16	0.063	1.24	3.13	3.54	3.88	0.098	0.81	99.63
**PES55**	Dubois	67.4	0.447	15.3	3.90	0.066	1.41	2.79	3.63	3.77	0.123	0.85	99.65
**PES62**	Petrelnuten	68.4	0.325	14.7	3.81	0.059	0.78	2.26	3.39	5.23	0.055	0.48	99.42
**PES63**	Petrelnuten	67.0	0.421	14.7	4.67	0.069	0.95	2.53	3.48	5.12	0.067	0.41	99.45
**PES64**	Petrelnuten	64.4	0.524	14.8	5.51	0.092	1.82	3.64	3.33	4.20	0.095	0.91	99.37
**PES65**	Petrelnuten	60.6	0.857	14.1	8.42	0.137	2.97	4.83	3.16	2.97	0.098	1.21	99.34

Table S9. continued.

Sample	Location	Ba [ppm]	Cr [ppm]	Ga [ppm]	Nb [ppm]	Ni [ppm]	Rb [ppm]	Sr [ppm]	V [ppm]	Y [ppm]	Zn [ppm]	Zr [ppm]
**PES36**	Lake 3	243	118	25	16	<10	149	78	84	30	82	510
**PES38**	Lake 3	247	89	27	22	14	133	105	124	46	117	290
**PES39**	Lake 3	227	72	25	23	17	109	123	171	45	130	448
**PES40**	Lake 3	272	63	24	12	<10	172	79	71	29	85	233
**PES47**	Lake 2	204	167	26	29	<10	171	56	14	49	112	796
**PES48**	Lake 2	167	209	24	17	<10	156	39	<10	28	70	488
**PES49**	Lake 2	257	109	27	29	<10	171	93	22	50	116	906
**PES50**	Lake 2	147	250	21	13	<10	157	30	16	20	51	299
**PES52**	Dubois	785	202	21	<10	24	116	366	73	28	77	214
**PES53**	Dubois	996	113	20	<10	<10	120	391	42	17	49	138

**PES54**	Dubois	840	129	20	<10	13	121	336	39	21	52	172
**PES55**	Dubois	785	144	19	<10	13	133	324	55	23	61	191
**PES62**	Petrelnuten	1751	180	21	23	12	122	267	30	36	89	338
**PES63**	Petrelnuten	1758	321	22	28	18	114	251	43	41	98	453
**PES64**	Petrelnuten	1361	327	23	22	24	113	289	75	43	104	433
**PES65**	Petrelnuten	861	318	22	36	30	97	265	110	61	158	333

Table S10. Aqueous concentrations of nutrients and simple organics as analyzed by Ion Chromatography (IC) in μgL^{-1}.

Sample	Location	NO_3^{2-}	PO_4^{3-}	SO_4^{2-}	Cl^-	F^-	Oxalate	Formic acid	Acetate	Propionate	Butyrate	Valerate
**PES 36**	Lake 3	1.24	<0.2	5.29	3.02	0.48	<0.2	<0.1	1.71	<0.1	<0.1	<0.1
**PES 38**	Lake 3	1.81	<0.2	7.66	10.38	0.35	<0.2	0.30	2.97	0.43	<0.1	<0.1
**PES 40**	Lake 3	2.25	<0.2	10.86	5.47	0.36	<0.2	0.23	6.91	0.34	<0.1	<0.1
**PES 47**	Lake 2	0.32	<0.2	5.18	4.25	0.40	<0.2	0.11	1.50	0.45	<0.1	<0.1
**PES 48**	Lake 2	0.10	<0.2	4.13	2.92	0.79	<0.2	0.19	1.88	0.48	<0.1	<0.1
**PES 49**	Lake 2	0.55	<0.2	8.64	9.29	0.59	<0.2	0.16	4.56	0.58	<0.1	<0.1
**PES 50**	Lake 2	1.43	<0.2	67.79	19.68	3.40	0.71	0.22	1.02	0.14	<0.1	<0.1
**PES 51**	Lake 2	1.33	<0.2	46.45	10.62	1.29	<0.2	0.12	1.13	0.13	<0.1	<0.1
**PES 52**	Dubois	0.14	<0.2	6.32	4.32	0.16	<0.2	0.12	0.98	0.37	<0.1	<0.1
**PES 53**	Dubois	0.30	<0.2	10.66	4.19	0.24	<0.2	0.21	2.47	0.71	<0.1	<0.1
**PES 54**	Dubois	<0.02	<0.2	12.87	5.88	0.13	<0.2	0.12	1.39	0.30	<0.1	<0.1
**PES 55**	Dubois	0.37	<0.2	11.44	1.60	0.22	<0.2	<0.1	1.45	0.20	<0.1	<0.1
**PES 42**	Petrelnuten	0.54	<0.2	1.56	1.17	0.20	<0.2	0.73	6.13	1.02	0.20	0.22
**PES 59**	Petrelnuten	0.28	<0.2	2.42	1.44	0.18	1.17	0.36	2.53	0.37	<0.1	<0.1
**PES 61**	Petrelnuten	2.30	0.85	5.35	1.51	0.34	1.61	0.36	17.37	0.35	0.15	<0.1
**PES 62**	Petrelnuten	0.55	2.09	5.86	1.23	0.22	1.88	0.13	1.22	0.17	<0.1	<0.1
**PES 64**	Petrelnuten	0.45	0.25	11.69	5.12	0.32	1.46	0.17	2.25	0.24	<0.1	<0.1

Supplementary Data S1: Output of the PCA of bacterial OTUs.

Partitioning of variance:

	Inertia	Proportion
Total	15747	1
Unconstrained	15747	1

Eigenvalues, and their contribution to the variance

Importance of components:

PC1 PC2 PC3 PC4 PC5 PC6 PC7 PC8 PC9 PC10 PC11 PC12 PC13 PC14 PC15 PC16

Eigenvalue 6793.2866 4678.3590 1.414e+03 1.028e+03 531.99097 404.11167 345.20355 191.92975 1.026e+02 76.465707 66.640577 56.009731 28.149296 12.864986 1.078e+01 5.5524155

Proportion Explained 0.4314 0.2971 8.976e-02 6.528e-02 0.03378 0.02566 0.02192 0.01219 6.516e-03 0.004856 0.004232 0.003557 0.001788 0.000817 6.847e-04 0.0003526

Cumulative Proportion 0.4314 0.7285 8.183e-01 8.835e-01 0.91733 0.94300 0.96492 0.97711 9.836e-01 0.988479 0.992711 0.996268 0.998056 0.998873 9.996e-01 0.9999101

	PC17
Eigenvalue	1.416e+00
Proportion Explained	8.991e-05
Cumulative Proportion	1.000e+00

Scaling 2 for species and site scores

* Species are scaled proportional to eigenvalues

* Sites are unscaled: weighted dispersion equal on all dimensions

* General scaling constant of scores: 24.26073

Species scores

	PC1	PC2	PC3	PC4	PC5	PC6
denovo1726	1.9330	–3.3075	–2.744973	0.89041	–0.3236	0.37377
denovo3345	–1.0710	–1.4295	–0.829714	0.63621	0.7122	–0.71516
denovo4865	–11.2220	–2.0645	–0.714512	1.61216	–0.2443	–0.82563
denovo8523	2.1145	4.3312	–0.002888	0.96140	0.1050	–0.88119
denovo10202	1.7369	5.8445	0.292209	1.57188	–0.3297	–1.62305
denovo12574	3.4820	–3.5348	–1.728445	–2.31705	–0.9573	–1.91509
denovo14041	–3.6746	–1.8151	–1.220601	–0.48451	2.1839	–0.68703
denovo15890	3.0874	–3.6962	–0.710738	2.29696	–2.5047	–0.50447
denovo15930	0.4346	–1.5190	–1.400352	–1.04964	0.4520	0.45420
denovo18602	1.2955	4.1246	–0.830046	0.88979	0.5602	–0.73865
denovo18695	1.6246	–2.4502	–0.934114	3.50553	0.5347	1.32931
denovo18789	0.4817	–2.6949	–1.881208	–1.20691	–0.2353	–0.37626
denovo19685	0.9268	0.1985	–1.592742	0.05793	0.9241	0.65926
denovo22765	5.5384	–6.0109	3.455035	1.07373	1.9540	–1.21702
denovo23995	2.3194	1.2102	–3.190476	1.61198	1.0131	–0.63293
denovo27012	1.9544	1.9546	–2.696771	–0.89699	0.7428	0.07036
denovo29111	–5.9147	–1.1008	0.212286	0.39085	–0.1575	–0.99265

Site scores (weighted sums of species scores)

	PC1	PC2	PC3	PC4	PC5	PC6
PES36	0.7064	3.98609	9.33174	–3.77326	–9.48330	14.451
PES38	3.2222	11.50566	0.98320	4.14821	0.60878	–6.651
PES39	3.5435	11.99882	1.24401	3.15030	–3.00226	–3.458
PES40	3.2832	10.67471	–2.71133	2.72923	2.49249	–1.338
PES42	–0.1608	1.91838	–5.16052	–4.91939	3.22027	7.447
PES43	–4.9232	0.56563	–0.01738	–4.43271	–3.60905	2.009
PES47	3.9929	–2.70408	–7.09890	–7.17259	–6.52145	–1.398

PES48	4.3767	–2.40723	–3.31813	–2.18144	–0.72628	2.237
PES49	5.1712	–2.69284	–1.03963	–8.71273	4.86474	–8.135
PES50	0.9759	–1.11788	–7.33151	–0.43796	7.69668	7.047
PES51	2.2320	1.69301	–7.18152	–3.67781	3.75658	4.116
PES52	4.4278	–5.55594	–0.98030	–1.45140	–13.66172	–6.453
PES53	4.3186	–5.28861	0.57451	8.09853	–3.05366	1.652
PES54	3.0778	–5.70140	–0.38226	7.32414	–1.69439	–2.447
PES55	2.4506	–4.75291	0.08320	10.05463	1.29713	1.652
PES56	1.2654	–4.71465	–0.95717	7.60960	5.74090	3.116
PES59	–8.7342	–0.05903	1.39786	0.16083	–0.45844	–1.557
PES60	–9.1794	–0.32675	0.86797	0.84275	0.09254	–2.726
PES61	–8.7210	–0.43970	–0.97533	–0.07724	0.42860	–2.707
PES62	–9.1640	–0.47268	–0.21848	1.86899	–1.24315	–1.905
PES63	–8.2342	–0.63115	–0.71051	–0.38146	0.36328	–1.311
PES64	2.7418	–3.69534	9.33742	–8.05294	7.12704	–5.731
PES65	3.3307	–1.78212	14.26309	–0.71629	5.76468	2.089

Supplementary Data 2: Output of the PCA of the eukaryotic OTUs.

Partitioning of variance:

Inertia Proportion

Total 15469 1

Unconstrained 15469 1

Eigenvalues, and their contribution to the variance

Importance of components:

PC1 PC2 PC3 PC4 PC5 PC6 PC7 PC8 PC9 PC10 PC11 PC12 PC13 PC14 PC15 PC16

Eigenvalue 3556.0027 3170.4533 2735.8550 1956.9294 1.135e+03 735.39034 664.64005 420.57004 323.2648 244.03926 161.29456 1.369e+02 1.168e+02 58.93167 35.539388 1.426e+01

Proportion Explained 0.2299 0.2050 0.1769 0.1265 7.339e-02 0.04754 0.04297 0.02719 0.0209 0.01578 0.01043 8.849e-03 7.551e-03 0.00381 0.002298 9.218e-04

Cumulative Proportion 0.2299 0.4348 0.6117 0.7382 8.116e-01 0.85915 0.90211 0.92930 0.9502 0.96598 0.97640 9.853e-01 9.928e-01 0.99661 0.998912 9.998e-01

PC17 PC18 PC19

Eigenvalue 2.1080097 4.405e-01 2.930e-02

Proportion Explained 0.0001363 2.848e-05 1.894e-06

Cumulative Proportion 0.9999696 1.000e+00 1.000e+00

Scaling 2 for species and site scores

* Species are scaled proportional to eigenvalues

* Sites are unscaled: weighted dispersion equal on all dimensions

* General scaling constant of scores: 23.58416

Species scores

	PC1	PC2	PC3	PC4	PC5	PC6
denovo630	0.21428	0.59621	–0.2311	0.486920	–1.566124	2.5029
denovo2262	0.28787	0.67569	0.6802	–0.526218	0.067123	0.2428
denovo5580	1.64674	0.61362	0.9724	–1.540197	0.169338	–0.3139
denovo6766	–0.21175	0.55173	0.2299	2.059635	–1.827537	2.4818
denovo7317	–1.51171	1.80307	–8.0330	–1.461693	1.864655	–0.1565
denovo7358	–0.02315	0.65206	0.6404	–0.353579	0.082539	0.2432
denovo9691	0.02972	1.38594	0.7367	1.592919	0.390766	0.3988
denovo9728	–8.38999	–4.83868	2.0418	–1.903572	–0.255986	–0.7586
denovo10529	1.73524	0.59058	1.0236	–2.146429	0.003463	–0.2367
denovo10938	2.95208	1.21416	1.7092	–3.762391	–0.042161	–0.2944
denovo12309	–1.23435	4.36815	3.1591	3.829199	2.896415	–1.3305
denovo13741	5.45465	–7.68878	–0.9051	2.347243	1.330446	–0.4851
denovo13822	–0.05318	1.08974	–1.5834	0.213848	–1.816457	–0.3682
denovo18417	0.70609	1.01138	–1.6878	1.647614	–3.979861	–3.0613
denovo21615	1.93243	0.42366	0.8629	–1.989633	0.096171	–0.3652
denovo27253	–0.42022	–0.53445	0.1142	0.001703	–0.509331	0.9990
denovo28736	–0.46569	0.03486	–1.5948	–0.489182	1.086853	0.2660
denovo29133	2.10434	0.88593	1.6992	–2.953475	–0.033885	–0.1408
denovo30251	–0.49077	–0.12643	–0.9470	–0.446115	0.734645	0.4070

Site scores (weighted sums of species scores)

	PC1	PC2	PC3	PC4	PC5	PC6
PES36	0.8848	4.2389	3.4155	–2.7643	0.17514	2.10197
PES38	–1.2094	9.8767	5.6640	8.1509	9.07234	–5.23181
PES39	–10.1635	–1.3921	4.9316	–2.5700	–0.62555	–3.56796
PES40	–10.1778	–1.7729	4.6998	–2.6251	–0.77559	–3.04725
PES42	–0.1265	8.2890	4.5611	10.5825	2.86929	3.02470

PES43	3.7570	–0.4121	–1.6422	6.0558	–11.86270	–10.83672
PES47	1.9263	–8.4653	0.7299	3.2262	1.78166	–0.56849
PES48	–8.3978	–3.6317	3.5739	–3.0306	–3.31781	0.67045
PES49	5.8685	–6.9895	–0.5386	5.5678	1.72984	2.41472
PES50	1.3569	–8.5337	0.6575	2.9057	2.49661	–1.81267
PES52	–1.6318	0.7390	0.4319	–0.7249	–0.53815	2.57969
PES53	–2.5479	1.8433	–10.1486	–3.0866	6.50475	2.17745
PES54	–2.7399	–4.2008	1.8805	0.1025	–3.96778	6.71029
PES55	–1.7059	3.2125	–11.6552	–2.6110	6.03782	0.77682
PES56	–2.7740	–3.9787	–5.9475	–1.5249	5.23123	–0.46331
PES59	7.7658	0.2901	2.9724	–6.7364	1.25232	–1.71041
PES60	6.3085	3.9234	4.4715	–9.9747	–0.62126	–0.78624
PES61	7.0495	2.9355	3.9362	–8.5098	0.05099	–1.14555
PES63	6.6609	–6.0380	–0.4312	3.9330	2.84985	–0.09898
PES64	0.6546	4.2428	–1.3518	2.9063	–9.02974	15.91727
PES65	–0.7583	5.8236	–10.2107	0.7278	–9.31328	–7.10397
